# Genetically engineered IgG1 and nanobody oligomers acquire strong intrinsic CD40 agonism

**DOI:** 10.1080/21655979.2024.2302246

**Published:** 2024-01-12

**Authors:** Nienke Hesen, Mohamed Anany, Andre Freidel, Mediya Baker, Daniela Siegmund, Olena Zaitseva, Harald Wajant, Isabell Lang

**Affiliations:** a Division of Molecular Internal Medicine, Department of Internal Medicine II, University Hospital Würzburg, Würzburg Germany; b Department of Microbial Biotechnology, Institute of Biotechnology, National Research Center, Giza, Egypt

**Keywords:** Antibody fusion protein, CD40, nanobody, valency, TNFRSF

## Abstract

Most anti-CD40 antibodies show robust agonism only upon binding to FcγR^+^ cells, such as B cells, macrophages, or DCs, but a few anti-CD40 antibodies display also strong intrinsic agonism dependent on the recognized epitope and/or isotype. It is worth mentioning, however, that also the anti-CD40 antibodies with intrinsic agonism can show a further increase in agonistic activity when bound by FcγR-expressing cells. Thus, conventional antibodies appear not to be sufficient to trigger the maximum possible CD40 activation independent from FcγR-binding. We proved here the hypothesis that oligomeric and oligovalent anti-CD40 antibody variants generated by genetic engineering display high intrinsic, thus FcγR-independent, agonistic activity. We generated tetra-, hexa- and dodecavalent variants of six anti-CD40 antibodies and a CD40-specific nanobody. All these oligovalent variants, even when derived of bivalent antagonistic anti-CD40 antibodies, showed strongly enhanced CD40 agonism compared to their conventional counterparts. In most cases, the CD40 agonism reached the maximum response induced by FcγR-bound anti-CD40 antibodies or membrane CD40L, the natural engager of CD40. In sum, our data show that increasing the valency of anti-CD40 antibody constructs by genetic engineering regularly results in molecules with high intrinsic agonism and level out the specific limitations of the parental antibodies.

## Introduction

Many preclinical and clinical studies, aimed at activating CD40 for tumor treatment or for vaccination against pathogens, use anti-CD40 antibodies [1–3]. With respect to the agonism of anti-CD40 antibodies, it is, however, extremely important to distinguish between antibody-intrinsic autonomous agonism, which takes place without binding of the antibody to Fcγ receptors (FcγRs), and a conditional type of CD40 agonism which becomes only apparent when the antibody has been bound to a cell expressed FcγR molecule. Thus, strictly and correctly speaking, anti-CD40 antibodies with FcγR-dependent agonism are no agonists, since their sole binding to CD40 does not have a receptor-stimulating effect. Rather, the complexes of these non-agonistic antibodies with other per se non-agonistic molecules (FcγRs, crosslinking antibodies, etc.) display the agonistic effect. Unfortunately, this distinction has often not been made in studies on CD40-targeting antibodies so that the term ‘agonist’ has been attributed in the literature to both types of anti-CD40 antibodies, such antibodies that only trigger relevant CD40 signaling when bound to FcγRs and such antibodies that can do this as ‘free’ molecules. The FcγR-dependency of agonistic activity, however, is of overwhelming relevance for the interpretation of data obtained with anti-CD40 antibodies *in vivo*.

The majority of the anti-CD40 IgG antibodies considered as agonistic antibodies in the literature, indeed, have no relevant intrinsic agonism and only act agonistic when bound to FcγRs or upon crosslinking with secondary antibodies or protein G [4]. For example, a mutant of the ‘agonistic’ anti-CD40-IgG1 ADC-1013 (Mitazalimab) harboring a N297Q mutation in the Fc part, which reduces glycosylation and FcγR binding, looses its ability to engage dendritic cells but displays restored agonism upon crosslinking with anti-human IgG [5]. Similarly, variants of the ‘agonistic’ anti-CD40 antibody APX005M (Sotigalimab) lack agonism upon removal of the Fc domain but display enhanced agonism on B cells upon introduction of the S267E mutation improving affinity for FcγRIIb [6]. Comparable results have also been reported for several other anti-CD40 antibodies including SEA-CD40 [7], G28.5 [8] and ChiLob-7.4 [9].

For a few anti-CD40-IgGs, however, significant FcγR-independent agonism has been reported. For example, Fab_2_ fragments of CP-870,893 (Selicrelumab) showed similar B cell activation as the corresponding complete antibody and secondary crosslinking of the latter showed no relevant further increase in activity [10]. Similar findings have been made with the anti-CD40 antibody CDX-1140 [11]. There is evidence that the isotype of an anti-CD40 antibody is of particular relevance for its agonistic activity. CP-870,893 and CDX-1140 are both of the IgG2 isotype and conversion of the anti-CD40-IgG1 ChiLob-4/7 into the IgG2 isotype comes along with significant FcγR-independent agonism [12]. Intriguingly, the agonistic activity of anti-CD40-hIgG2 antibodies could be assigned to isoform B of the hIgG2 isotype, which differs from the A isoform of the hIgG2 molecule in the formation of the disulfide bridges between the CH1 domain and the CL and hinge domains, and which has thus a less flexible arrangement of the two Fab domains of the molecule [13–17]. Anti-CD40-hIgG2 antibodies that have mutations that instruct only the formation of isoform A (e.g. HC-C233S) or isoform B (e.g. HC-C127S or LC-C214S/HC-C233S) have therefore no or increased FcγR-independent agonism compared to the parental hIgG2 molecule [12,17,18]. However, the general relevance of IgG2 isotype and the IgG2B isoform for FcγR-independent intrinsic CD40 agonism is not undisputed and has been challenged by elaborate in vivo studies with human FcγR knock-in mice [19]. Moreover, the FcγR-independent CD40 response of anti-CD40-hIgG2 or anti-CD40-hIgG2B antibodies, which sometimes are called ‘superagonists,’ is still significantly lower than the agonism of FcγR-bound anti-CD40 antibodies [19,20].

It is worth to mention that preclinical studies and clinical trials with FcγR- and C1q-interacting anti-CD40 antibodies give evidence for significant dose limiting toxicity (cytokine release syndrome (CRS), hepatotoxicity) [2,3,21–23], while clinical studies with anti-CD40 antibodies lacking Fc effector functions showed excellent tolerability [24–27]. The higher toxicity of FcγR/C1q-interacting versus Fc-silent anti-CD40 antibodies suggests that FcγR-bound anti-CD40 antibody molecules and/or complement activation cause the dose-limiting activities and not the free anti-CD40 antibody molecules. Furthermore, prototypic targets of CD40 signaling, such as TNFα, IFNγ, and IL-12p40, have been identified as effector molecules mediating the toxic effects of FcγR-interacting anti-CD40 antibodies [21,23]. Together, these findings suggest that CD40 activation crucially contributes to the dose-limiting toxicity of FcγR-/complement-activating anti-CD40 antibodies and that FcγR-independent CD40 agonists will also have this limitation. The therapeutic use of potent intrinsically agonistic CD40 antibodies may need, therefore, special therapy regimens localizing the antibody activity to the tumor, e.g. by intratumoral application [28–32].

Here, we proved the hypothesis that oligomeric and oligovalent anti-CD40 antibody variants display high intrinsic, thus FcγR-independent, agonistic activity. We found i) that the enforced stoichiometric oligomerization of anti-CD40 molecules by genetic engineering regularly confers strong FcγR-independent agonism and ii) that the recognized epitope of an anti-CD40 antibody has typically no major effect on the intrinsic agonism of oligovalent anti-CD40 variants. In accordance with the central role of valency for the agonism of CD40 antibodies, we further showed that hexa- and nonavalent constructs of a CD40-specific nanobody display much higher CD40-stimulatory activity than bi- and tetravalent nanobody constructs.

## Results

### FcγR-bound anti-CD40 antibodies uniformly display strong agonism irrespective of isoform and the epitope recognized

**Figure 1.**
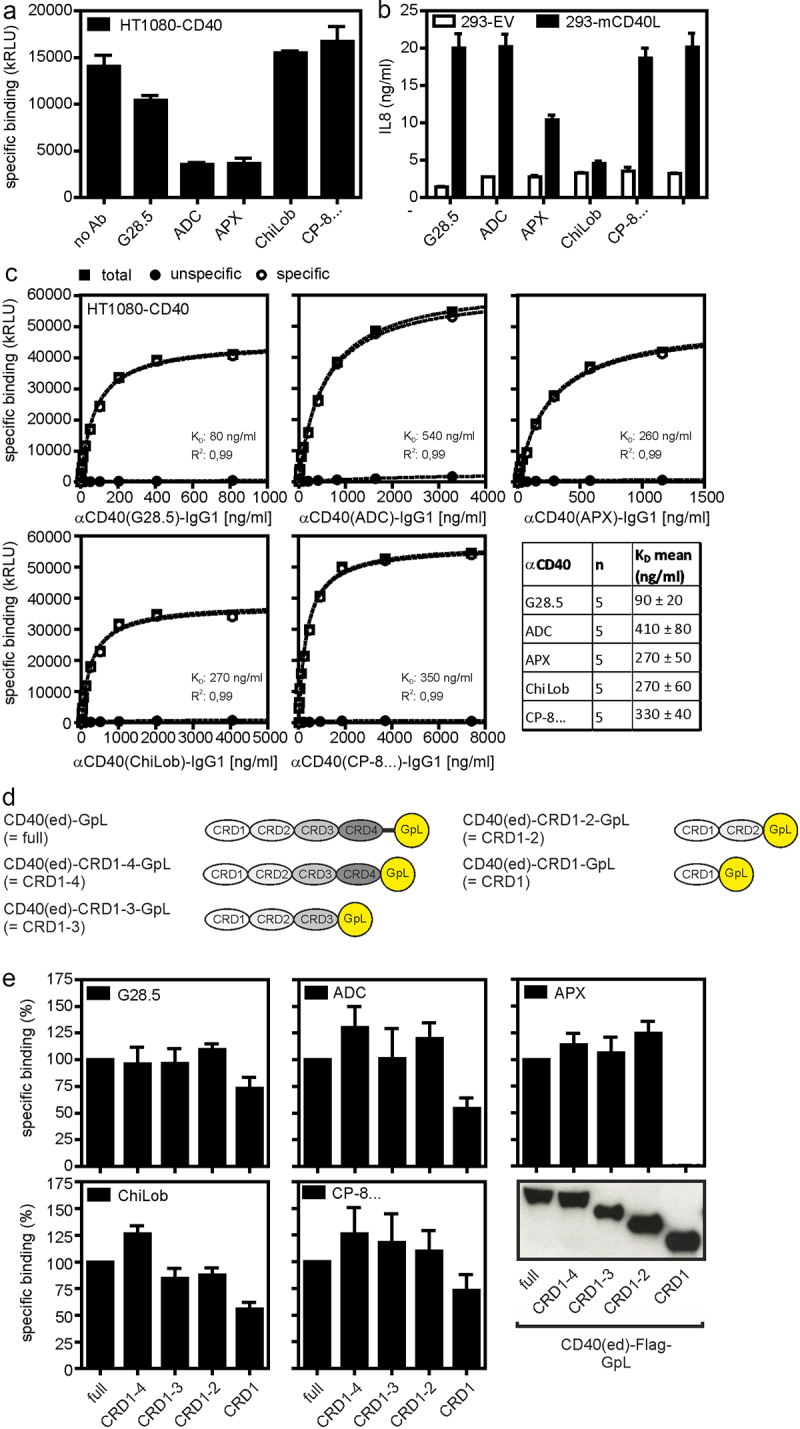
Characterization of anti-CD40 antibodies. (a) HT1080-CD40 cells were preincubated with 5 μg/ml of the indicated anti-CD40 antibodies or remained untreated (no ab) and then binding of GpL-TNC-CD40L (100 ng/ml) was determined. (b) U2OS cells were treated with 10 µg/ml of the indicated antibodies along with HEK293 transfectants expressing memCD40L or empty vector. Next day, CD40-induced IL8 production was evaluated by ELISA. (c) Specific binding of anti-CD40-IgG1-GpL fusion proteins to HT1080-CD40 transfectants. Diagrams show results from one of five binding experiments for each protein and the table lists the averaged K_D_-values derived of five independent experiments. (d) Domain architecture of CD40-GpL and CD40-GpL deletion mutants used in E. (e) Protein G-coated ELISA plates were loaded with anti-CD40 antibodies. Protein G/anti-CD40 antibody complexes were finally incubated with C-terminal deletion mutants of the CD40 ectodomain harboring a C-terminal *Gaussia princeps* luciferase (GpL) reporter domain (see western blot, lower panel, left) or TNFR2(ed)-GpL as negative control. Specific binding of the CD40 deletion mutant molecules were finally obtained by the subtraction of the unspecific TNFR2(ed)-GpL binding values from the total binding values of the various CD40-GpL fusion proteins.

In view of the complex and sometimes even contradictory findings on the FcγR-dependency of the agonism of anti-CD40 antibodies, we initially defined a panel of anti-CD40 antibodies with widely differing properties. This panel included the well-established and long known anti-CD40 antibody G28.5 [33] as well as several anti-CD40 antibodies currently being investigated in clinical trials and/or disclosed in patents (CP-870,893/Selicrelumab [34] APX005M/Sotigalimab [6] ADC-1013/Mitazalimab [5] ChiLob7.4 [20]). All antibodies of the panel recognize the human CD40 molecule, but differ in their effect on CD40L binding. IgG1 variants of ADC-1013, APX005M, and G28.5 strongly or partly inhibited CD40 binding of GpL-TNC-CD40L (Figure 1a), a *Gaussia princeps* luciferase (GpL) fusion protein of soluble trimeric CD40L stabilized by the trimerization domain of tenascin-C (TNC). CP-870,893-IgG1 and ChiLob7.4-IgG1 did not affect GpL-CD40L binding to CD40 (Figure 1a). In line with this, the latter two antibodies had no effect on production of IL8 induced by cells expressing membrane CD40L (memCD40L), the natural stimulator of CD40. IL8 is an easily measurable chemokine whose expression is dominantly regulated by the classical NFκB signaling pathway and which is thus efficiently produced in response to CD40 engagement (Figure 1b). In contrast, ADC-1013-IgG1 and APX005M-IgG1 significantly inhibited this CD40 response (Figure 1b). G28.5-IgG1, which only partly blocked GpL-CD40L binding of CD40, also showed no inhibitory activity (Figure 1b). The antibodies of the panel furthermore differed in CD40 affinity and with regard to the region in the extracellular domain of CD40 with which they interact (Figure 1(c–e)). Cellular equilibrium binding studies with variants of the various anti-CD40 antibodies with a GpL reporter domain genetically fused to the C-terminus of the light chain resulted in affinities for cell-expressed CD40 between 90 and 410 ng/ml (Figure 1c). Binding studies with deletion mutants of the CD40 extracellular domain lacking one to four of the characteristic cysteine-rich domains (CRDs) of CD40 (Figure 1d) revealed furthermore that the antibodies bind to CRD1 or CRD2 (Figure 1e).

**Figure 2.**
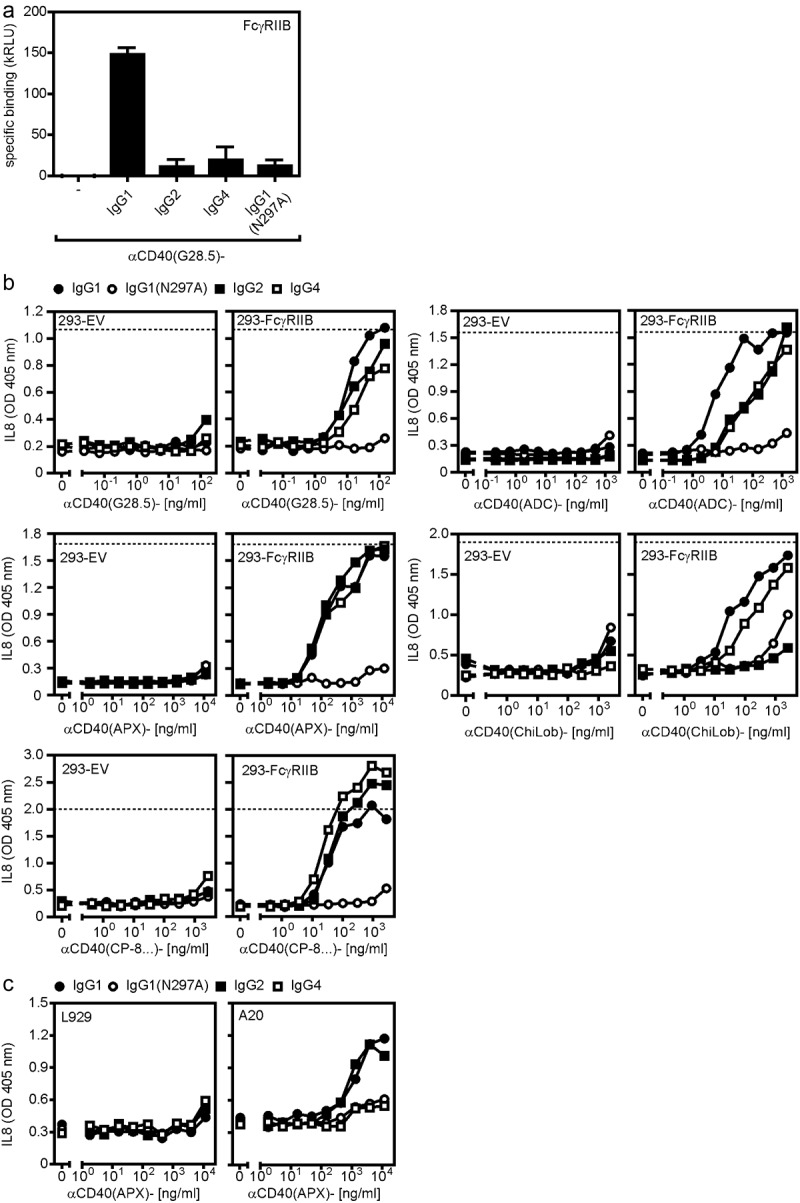
FcγR binding boosts anti-CD40 antibody-triggered IL8 production. (a) Specific binding of IgG1, IgG2, IgG4 and IgG1(N297A) variants of G28.5 to murine FcγRIIB. (b) CD40-responsive U2OS were challenged with IgG1, IgG2, IgG4 and IgG1(N297A) variants of the indicated anti-CD40 antibodies along with HEK293 cells transfected with empty vector (EV) or expression plasmids encoding FcγRIIB. Next day, cell supernatants were analyzed for IL8 production as readout of CD40 activation. (c) Co-cultures of U2OS and murine A20 cells, which endogenously express FcγRs, were stimulated with the αCD40(APX)-IgG antibodies and the next day, IL8 production was again determined by ELISA.

**Figure 3.**
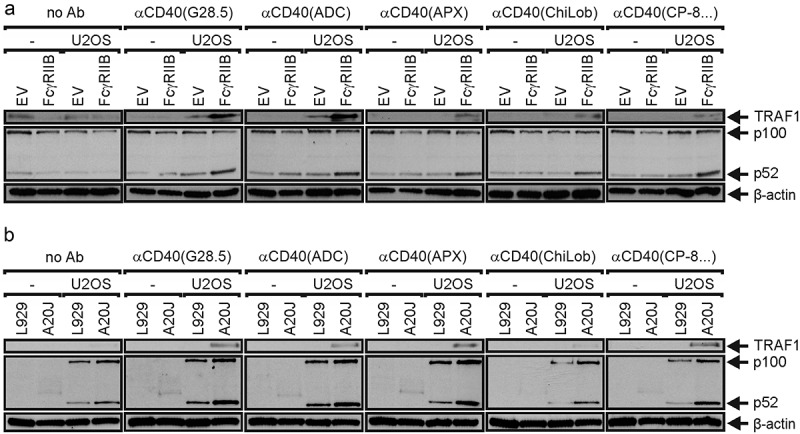
FcγR binding enhances anti-CD40 antibody-induced p100 processing. (a) U2OS cells were stimulated overnight with HEK293 cells transfected with empty vector (EV) or an expression plasmid encoding FcγRIIB and 150 ng/ml of the indicated antibodies or remained without antibody treatment (no ab). Total cell lysates were analyzed for p100 processing. (b) Human U2OS cells were stimulated overnight with murine L929 cells not expressing FcγRs or murine A20 cells expressing endogenously FcγRIIB and again 150 ng/ml of the indicated antibodies. L929 and A20 cells were also antibody-treated in the absence of U2OS cells (-) as control for bands no derived of the human U2OS cells. Total cell lysates were again analyzed for p100 processing.

The ‘agonistic’ anti-CD40 antibodies which are currently in clinical development are IgG1, IgG2 or IgG4 antibodies. The different *in vitro* agonism of these antibodies as well as their different *in vivo* tolerability has been attributed, at least in part, to isotype differences [3,35]. We therefore cloned the variable domains of the anti-CD40 antibodies of our panel not only in their original isotype, but also in the other two of the three isotypes mentioned. In addition, we also generated IgG1 variants (IgG1(N297A)) of the antibodies carrying a point mutation (N297A), which strongly reduces binding to FcγRI and abrogates binding to the remaining FcγRs [36]. First, we evaluated the ability of the conventional IgG variants of the anti-CD40 antibody panel to induce FcγR-dependent CD40 activation. For this purpose, we used a simple *in vitro* system, in which CD40-expressing responder cells secreting large amounts of IL8 after CD40 stimulation (U2OS cells or HT1080-CD40 transfectants) were co-cultivated with HEK293 cells. The latter express little IL8 and were transiently transfected with the murine FcγRIIB capable of binding IgG1 and weakly IgG2 and IgG4 (Figure 2a), or with an empty vector (EV). The use of FcγR transfectants had three advantages over the use of cells with endogenous FcγR expression. First, it was possible to analyze one type of FcγR without the possible interference by other FcγR types. Second, there was a perfect negative control namely EV transfected HEK293 cells and third the high expression levels reached by transient transfection minimized the risk that CD40 engagement by FcγR-bound anti-CD40 antibodies is underestimated simply by the fact that the number of CD40 molecules in the system exceeds the number of FcγRs available for anti-CD40 presentation. All wild-type anti-CD40 IgG variants with the exception of those of the antibody ChiLob7.4, efficiently stimulated the production of IL8 in co-cultures with HEK293 transfectants expressing FcγRIIB reaching a similar or almost the same maximum response as obtained with memCD40L transfectants (Figure 2b). The agonistic activity of the IgG1 and IgG4 ChiLob7.4 variants was still significantly increased in co-cultures with FcγRIIB expressing HEK293 cells but not fully reached the level of memCD40L. In striking contrast, in co-cultures with HEK293 EV transfectants, we found no or only a significantly weaker IL8 induction and only at high antibody concentrations. As expected, the anti-CD40 IgG1(N297A) mutants of the various antibodies that do not bind to FcγRIIB showed little or no agonism both in co-cultures with FcγRIIB-transfected HEK293 cells (Figure 2b) as well as in co-cultures with EV transfectants (Figure 2b). Generally similar results were obtained for the IgG1 and IgG4 variants using cocultures with CD40 responder cells and murine A20 cells expressing FcγRs endogenously (Figure 2c). A similar response pattern as for anti-CD40 antibody-induced IL8 production was also observed when CD40-mediated activation of the alternative NFκB signaling pathway was investigated. The alternative NFκB pathway essentially activates a different group of NFκB transcription factors than the classical one and uses for this a distinct signaling pathway [37]. Activation of the alternative NFκB pathway is characterized by the processing of a precursor protein termed p100 to the NFκB transcription factor subunit p52. In co-cultures of FcγRIIB-expressing HEK293 transfectants and U2OS cells, all anti-CD40 antibodies were able to efficiently trigger p100 to p52 processing and to upregulate expression of the TRAF1 protein, which is induced by both NFκB signaling pathways (Figure 3). In contrast, in co-cultures with HEK293 control transfectants, there was only little or no evidence for activation of the alternative NFκB signaling pathway. Together, these results confirmed previous studies [18–20,38] reporting that the agonism of plasma membrane-presented complexes of anti-CD40 antibodies and FcγRs is regularly much higher than that of the anti-CD40 IgG antibodies, irrespective of the epitope recognized by a particular anti-CD40 antibody and its isotype. Moreover, since the maximum IL8 induction achieved by FcγR-bound anti-CD40 antibodies was comparable to the IL8 production induced by membrane CD40L expressing cells, it appears that FcγR-bound anti-CD40 antibodies, but not ‘free’ anti-CD40 antibodies are able to stimulate the maximum possible CD40 activity.

### Genetically engineered anti-CD40 antibody oligomers are strong agonists

**Figure 4.**
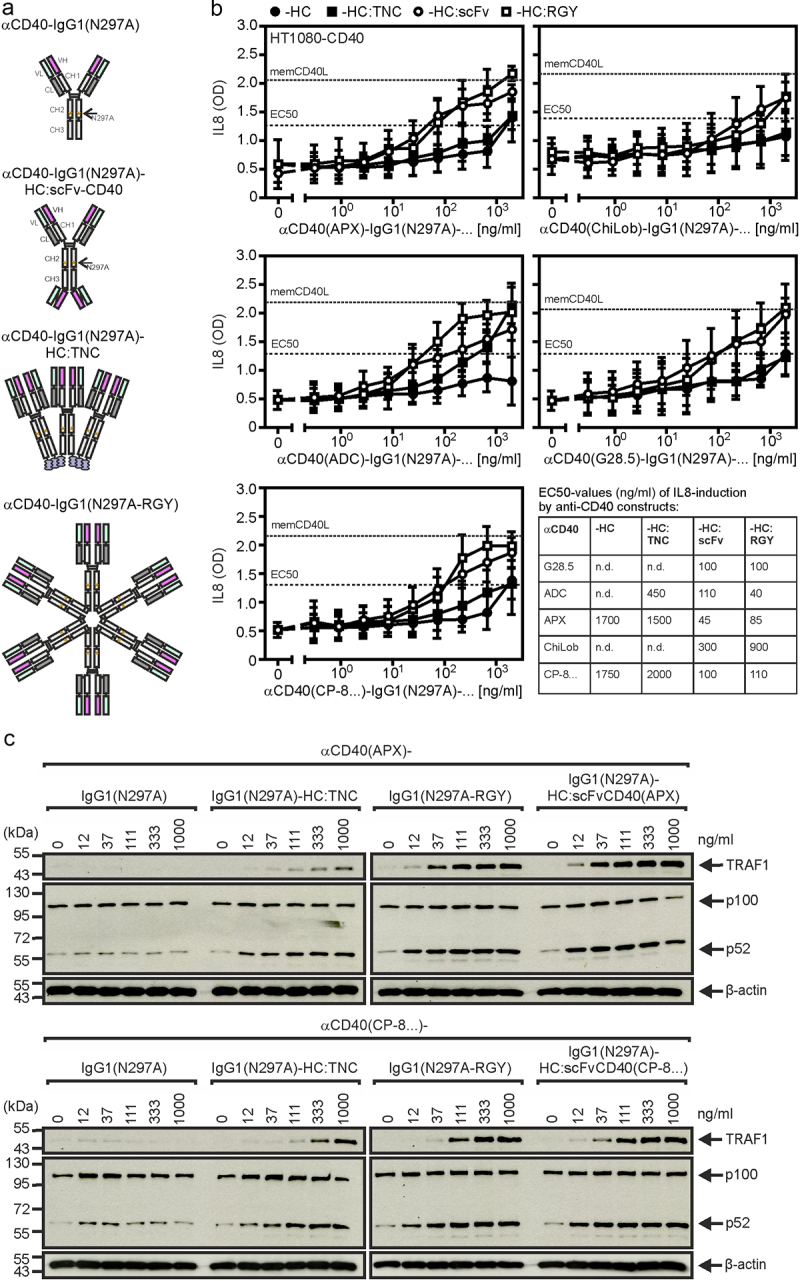
Oligovalent anti-CD40-IgG1(N297A) variants and anti-CD40-IgG1(N297A)-HC:scFvCD40 fusion proteins exert FcγR-independent CD40 agonism. (a) Domain architecture of the genetically engineered oligomerized anti-CD40-IgG1(N297A)-TNC and anti-CD40-IgG1(N297A-RGY) antibody variants and the tetravalent anti-CD40-IgG1(N297A)-HC:scFvCD40 fusion proteins. (b) HT1080-CD40 cells, which strongly produce the NFκB-regulated cytokine IL8 after CD40 stimulation, were stimulated overnight with the different anti-CD40 antibody variants and finally the IL8 production was recorded by ELISA. HT1080-CD40 cells were also challenged with membrane CD40L transfected HEK293 cells. The resulting IL8 production was defined as maximal and used to define the possible half-maximal IL8 response. Shown are the mean values derived of 5–6 independent experiments. (c) U2OS cells were challenged with the indicated concentrations of the different antibody variants of anti-CD40(APX) and anti-CD40(CP-8 …) overnight. Next day total cell lysates were analyzed for p100 processing.

Since it is well established that crosslinking of anti-TNFR antibodies with secondary antibodies or protein A or protein G can enhance their agonistic activity [4], we investigated whether the defined oligomerization of anti-CD40 antibodies by genetic engineering results in agonistic molecules. For this purpose, we generated trimeric, thus hexavalent, and hexameric, thus dodecavalent variants of all IgG1 (N297A) antibodies of our anti-CD40 antibody panel. The hexavalent antibody variants were obtained by genetic fusion of the trimerization domain of the tenascin-C molecule, comprising about 30 amino acids, to the C terminus of the heavy chain of the IgG1 (N297A) molecules (Figure 4a). The dodecavalent antibody variants were obtained by introducing point mutations (RGY) into the IgG1 (N297A) heavy chain [39], which promote the assembly of hexameric IgG1 molecules (Figure 4a). The various anti-CD40-IgG1 (N297A)-TNC variants induced at concentrations between 0.2 and 2 µg/ml the half maximal IL8 response of the benchmark memCD40L expressing cells (Figure 4b). The anti-CD40-IgG1 (N297A-RGY) variants also acted as efficient CD40 agonists and were even regularly somewhat more efficient in CD40-dependent IL8 induction than the anti-CD40-IgG1 (N297A)-TNC variants (Figure 4b). In fact, with exception of the ChiLob7.4-based variant, all anti-CD40-IgG1 (N297A)-RGY constructs induced half maximal CD40-dependent IL8 production at concentrations below 100 ng/ml (Figure 4b). We also examined tetravalent variants of the anti-CD40-IgG1 (N297A) antibodies which were obtained by fusing scFv domains derived from the variable domains of the antibodies to the C-termini of the antibody heavy chains (Figure 4b). These IgG1 (N297A)-HC:scFv variants also showed a strong agonistic effect for all CD40 antibodies and were comparable active as the anti-CD40-IgG1 (N297A)-RGY variants. We also analyzed p100 processing and TRAF1 induction for the variants of the blocking anti-CD40 APX005 and the non-blocking anti-CD40 CP-870,893. While the conventional IgG1 (N297A) variants of both antibodies showed no (APX0005) or an at best very weak (CP-870,893) effect on p100 processing and TRAF1 induction, the two oligomerized antibody forms and the tetravalent variants triggered this response efficiently starting at concentrations below 100 ng/ml (Figure 4c). Again, the anti-CD40-IgG1 (N297A)-TNC variants showed a somewhat reduced efficacy compared to the anti-CD40-IgG1 (N297A-RGY) and anti-CD40-IgG1 (N297A)-HC:scFv variants. The latter two variants of the anti-CD40 antibody CP-870,893 fully remained their agonistic activity even after 1 week of incubation at 37°C suggesting that these two antibody formats are comparably stable as conventional antibodies (Supplemental Figure 1a,b).

The data shown so far were obtained with cell culture supernatants containing the anti-CD40 variant of interest. To verify that i) the agonistic activities observed are indeed related to oligomerized antibody molecules and that ii) the anti-CD40 fusion proteins maintain integrity, we exemplarily purified the CP-870,893 variants by gravity flow affinity chromatography on an anti-Flag antibody M2 agarose (Supplemental Fig. S2A). In fact, gel filtration analysis revealed peaks corresponding to the oligomerized antibody molecules, but in the case of CP-870,893-IgG1 (N297A)-RGY and CP-870,893-IgG1 (N297A)-TNC, there were also peaks corresponding to the parental non-agonistic non-oligomerized antibody molecules (IgG, Supplemental Fig. S2B). Most important, the purified proteins were still efficient CD40 agonists (Supplemental Fig. S2C). Therefore, although the fraction of non-oligomerized non-agonistic parental antibody IgG1 (N297A) molecules might lower the specific activity of the CP-870,893-IgG1 (N297A)-RGY and CP-870,893-IgG1 (N297A)-TNC samples a bit, the purified samples were useful for follow-up analysis of the agonism of the oligomerized molecule species. Worth mentioning, the IgG1 version of CP-870,893 but not its IgG1 (N297A) variant stimulated IL8 production in immature dendritic cells (iDCs) derived of human monocytes confirming again the agonism of FcγR-bound CP-870,893-IgG1 and the efficacy of the N297A mutation to prevent agonism-conferring FcR binding (Supplemental Fig. S2D). Importantly, the oligomerized variants of CP-870,893-IgG1 (N297A) were again able to stimulate IL8 production in iDCs derived of human monocytes, confirming their autonomous molecule-intrinsic agonistic activity (Supplemental Fig. S2D).

### Genetically engineered oligovalent variants of a CD40-specific single domain antibody are strong agonists

**Figure 5.**
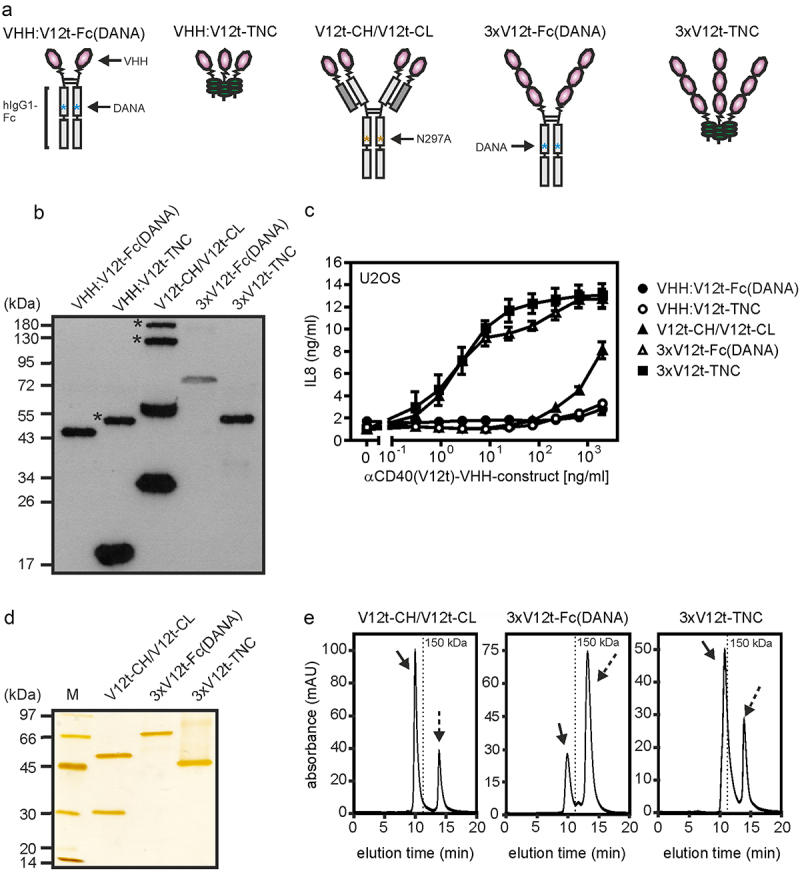
Oligomeric sdAb:cd40 variants display strong CD40 agonism. (a) Domain architecture of oligomeric sdAb:CD40 variants. DANA refer to the D256A-N297A mutation preventing/reducing FcγR binding of IgG1. (b) Western blot analysis of reduced sdAb:cd40 variants. Position of MW (kDa) markers are indicated. Asterixes refer to not fully reduced protein species. (c) U2OS cells were stimulated overnight with the different VHH:CD40 variants and finally IL8 production was determined by ELISA. (D,E) the indicated constructs were purified by affinity chromatography and analyzed by SDS-PAGE (D) and gel filtration (E). Dotted arrows indicate flag peptide remained from the affinity purification.

**Figure 6.**
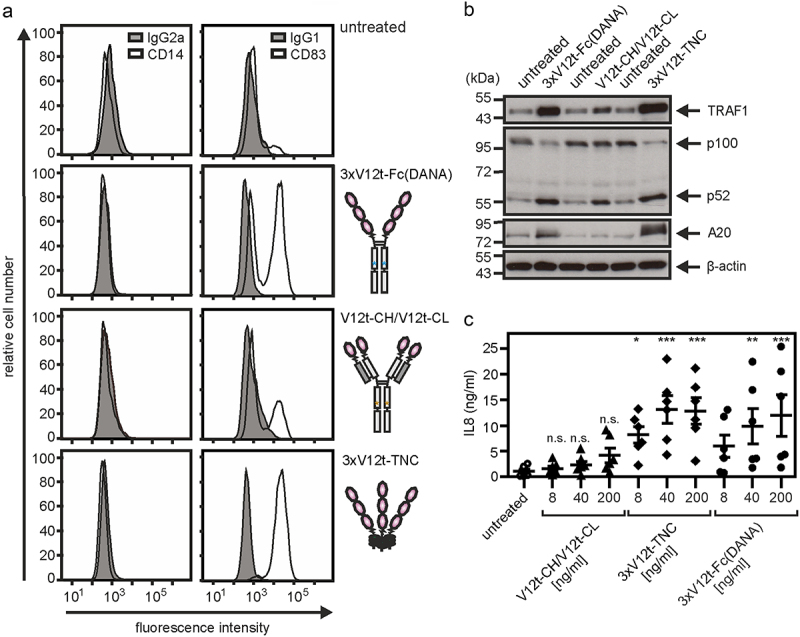
CD40 agonists with intrinsic agonism trigger maturation and activation of DCs. immature monocyte-derived dendritic cells (iDcs) were generated by cultivation of monocytes for 7 days with GM-CSF/IL4. (a) iDCs were treated with 200 ng/ml of the indicated constructs and were then analyzed after 2 days by flow cytometry for the cell surface expression of CD14 (left panel) and CD83 (right panel). (b) iDCs were treated with 200 ng/ml of the indicated constructs and were analyzed next day by western blotting for the presence of the indicated proteins. (c) iDCs were treated with 8, 40 or 200 ng/ml of the indicated constructs overnight and finally cell culture supernatants were analyzed for their IL8 content by ELISA. n.s. = non significant; *p < 0.05; **p < 0.01, ***p < 0.001.

Our data suggest that the number of CD40 binding sites within a CD40-targeting antibody construct is the prime factor that decides about the autonomous agonism of the construct rather than the characteristics (affinity, recognized epitope, blocking/non-blocking) and structural nature of its CD40 binding domains (Fab, scFv). To further verify this idea, we generated and analyzed oligovalent constructs composed of the human CD40-specific single-domain antibody (VHH) V12t [40] and oligomerizing protein scaffolds (Figure 5a,b). Similar to conventional bivalent anti-CD40 IgG antibodies, a bivalent VHH:V12t-Fc and a trivalent VHH:12-TNC fusion protein proved to be poorly active on the CD40^+^ U2OS cell line (Figure 5c). However, when three VHH:V12t domains were cloned sequentially to the Fc domain of human IgG1 (N297A) or to the trimerization domain of TNC, hexavalent and nonavalent molecules (3xV12-Fc (DANA), 3xV12-TNC) were obtained which show strong CD40-stimulating activity even higher than those of the various anti-CD40 antibodies in the anti-CD40-IgG1 (N297A).HC:scFv format (Figure 5c compares with Figure 4b). A tetravalent VHH:V12t variant which was obtained by replacing the variable domains of an IgG1 molecule with the VHH:V12t domain (V12t-CH/V12t-CL) showed also minor activity but was by far less active (Figure 5a–c). Gel filtration analysis of the affinity purified oligovalent constructs showed homogenous molecule species for all three constructs (Figure 5d,e). In accordance with the data obtained with the CD40^+^ U2OS cell line, 3xV12t-Fc (DANA) and 3xV12t-TNC efficiently triggered maturation of monocyte-derived iDCs, stimulated processing of p100 to 52 and induced expression of the NFκB targets TRAF1, A20 and IL8 (Figure 6a–c). The tetravalent V12t-CH/V12t-CL variant was again poorly active (Figure 6a–c). The 3xV12t-Fc (DANA) variant was also analyzed for stability by incubation at 37°C for up to 1 week and revealed no evidence for instability (Supplemental Fig. S3A,B).

To definitely proof finally that the intrinsic agonism of the anti-CD40-IgG1 (N297A)-HC:scFv antibody format, which we favored due to its very low aggregate content, and the oligomeric 3xV12-TNC variants indeed arise from the correctly assembled molecule species and not from a very small amount of nonspecifically aggregated highly active molecules, we purified the correctly assembled molecule species from anti-CD40 (G28.5)-IgG1-HC:scFv (G28.5), anti-CD40 (Apexi …)-IgG1-HC:scFv (Apexi …), anti-CD40 (CP-8 …)-IgG1-HC:scFv (CP-8 …) and 3xV12-TNC by anti-Flag affinity chromatography and subsequent preparative gel filtration. The molecules derived from the Fc-domain assembled homodimer peak of the three anti-CD40-IgG1 (N297A)-HC:scFv variants and of the TNC-domain assembled homotrimer peak of 3xV12-TNC were highly active (Supplemental Figure 4a,b). Again, the nanobody-based variant outperformed the antibody-based constructs with respect to the EC50 value (Supplemental Figure 4a,b).

## Discussion

In the past decade, extensive *in vitro* and *in vivo* studies have revealed that virtually every CD40-specific antibody displays agonistic activity if it is bound to FcγRs [8,19,20,41–43]. As improving FcγR-binding of antibodies by genetic engineering is state of the art, it appears trivial to achieve strong CD40 agonism *in vivo* by help of anti-CD40 antibodies with strong conditional FcγR-dependent agonism. The need for FcγR-binding, however, comes along with several limitations. The expression levels of CD40 are typically quite high and FcγR-expressing immune cells can be of limited availability. Thus, one has to consider that *in vivo* only a fraction of CD40 molecules can be targeted by FcγR-bound anti-CD40 antibodies resulting in submaximal stimulation of CD40 activities. For example, anti-CD40 murine IgG1 antibodies significantly stimulate proliferation of B cells from wild-type mice but not of B cells derived of FcγRIIB-deficient mice [20]. However, if FcγR-expressing transfectants were added to the FcγRIIB-deficient B cells, proliferation increased 10- to 100-fold compared to the anti-CD40 antibody treated wild-type B cells [20]. This suggests that the levels of FcγRs expressed by B cells are too low to ensure that all CD40 molecules are targeted by FcγR-bound anti-CD40 antibodies. Another aspect that limits the utility of the FcγR-dependent agonism of conventional anti-CD40 antibodies for immune stimulation is the triggering of FcγR immune effector functions, e.g. ADCC, CDC, and/or ADCP. In such cases, FcγR-binding of anti-CD40 antibodies not only results in CD40 agonism but also in the killing of the CD40-expressing target cell [44]. Anti-CD40 antibody variants with robust autonomous, thus molecule-intrinsic and FcγR-independent agonism can obviously overcome these limitations and could open new avenues in the exploitation of CD40 as a therapeutic target.

Interestingly, the situation regarding the agonism of anti-CD40 antibodies is very similar to that of the CD40-stimulating activity of soluble CD40L. The latter is a soluble trimeric molecule released by proteolytic processing from trimeric membrane CD40L. While membrane CD40L efficiently stimulates CD40 signaling, the CD40-stimulating abilities of soluble CD40L trimers are much weaker. However, physical connection of two or more soluble CD40L molecules by genetic fusion with appropriate oligomerization domains results in highly active molecules comparable active to membrane CD40L [45–49]. Moreover, there is growing evidence that receptor clustering is a crucial issue for activation of receptors of the TNFRSF including CD40 [50]. We, therefore, evaluated in this study the hypothesis that genetically engineered oligomers of anti-CD40 antibodies or, alternatively, anti-CD40-IgG molecules equipped with additional CD40 binding sites acquire a high CD40-stimulatory activity independent from FcγR-binding. For this purpose, we analyzed tetra-, hexa- and dodecavalent variants of six independently developed anti-CD40 antibodies differing in their effect on CD40L binding and affinity along with various oligomeric variants of a CD40-specific nanobody. In contrast to IgG1, IgG2, and IgG4 variants of these antibodies, which only displayed strong CD40 agonism when bound to FcγRs (Figures 2 and 3), all these variants engaged CD40 to a similar extent as memCD40L expressing cells at concentrations well below 1 µg/ml (Figure 4). Similarly, hexa- and nonavalent VHH:CD40 constructs but not bi- and trivalent variants showed potent CD40 agonism (Figures 5 and 6). Intriguingly, two of the parental anti-CD40 antibodies used were antagonistic anti-CD40 antibodies (Figure 1a,b). Thus, increasing the oligomeric state or the valency of anti-CD40 antibodies obviously overrules epitope-specific effects on CD40 activity. In sum, our results suggest that increasing valency is a generic design principle which allows to use practically any anti-CD40 antibody derived CD40 binding domain (Fab, scFv, VHH) to construct CD40-specific agonists with high intrinsic activity. Such antibody-based CD40 agonists are especially interesting for applications aiming on the sole and exclusive stimulation of CD40 signaling without triggering of FcγRs. The major potential therapeutic aim which could be preferentially achieved with these types of CD40 antibody constructs is certainly the stimulation of antigen-presenting cells to improve vaccination or to booster anti-tumor responses. FcγR-independent authentic CD40 agonists, as described in this study, however, are not useful when CD40 targeting is envisaged with the intention to stimulate FcγR functions, such as ADCC, ADCP, and CDC to kill tumor cells with high CD40 expression levels [44]. Worth mentioning, genetic engineering of the Fc domain of CD40, to achieve lack of FcγR binding or to reach preference for the binding of the inhibitory FcγRIIB, enables the empowerment of anti-CD40 antibodies to act together with soluble CD40L as agonists or as conditional CD40 agonists with FcγRIIB-restricted agonism [44]. Thus, the choice to use an authentic anti-CD40 agonist as described in this study or a certain type of conventional anti-CD40 antibody is highly dependent on the concrete therapeutic aim.

## Material and methods

### Cell culture

HEK293, U2OS, L929, A20J, and HT1080 cells (ATCC, Rockville, MD, USA) as well as HT1080-CD40 transfectants [49] were cultivated at 37°C and 5% CO_2_ and were regularly split twice a week 5–8-fold. HEK293, A20J, and HT1080/HT1080-CD40 cells were cultivated in RPMI 1640 medium (Sigma-Aldrich, Steinheim, Germany) and U2OS and L929 cells in DMEM with high glucose medium (Sigma-Aldrich, Steinheim, Germany). Culture media were supplemented with 10% fetal calf serum (FCS; Life Technologies, Karlsruhe, Germany).

### Expression plasmids

To obtain expression plasmid for membrane CD40L the corresponding full-length DNA sequence was cloned into the pEYFP-C1 vector. FcγRIIB expression plasmid (pCMV-SPORT6) was obtained from SourceBioScience (Nottingham, UK). Standard cloning techniques, DNA amplicons, and synthetic DNA fragments were used to generate pCR3-based expression plasmids encoding the proteins and antibody chains listed in Supplemental Table S1. The antibody variants used in this study and the plasmids(s) used for their production are listed in Supplemental Table S2. The sources of amino acid sequences used are listed in Supplemental Table S3.

### Production and purification of recombinant proteins

All recombinant proteins were produced by transfection of HEK293T cells with corresponding expression plasmids using polyethylenimine (PEI; Polyscience Inc., Warrington, USA) as described elsewhere in detail [51]. For the generation of recombinant antibody variants comprising a light and a heavy chain, a 1:1 mixture (mass ratio) of the corresponding expression plasmids was used. Five to seven days post-transfection, cell culture supernatants of transfected cells were collected and cleared from cell debris by centrifugation for 10 min (4°C, 4630 × g). The concentration of the recombinant proteins was estimated by western blot analysis of the supernatants along with Flag-tagged protein standards of known concentrations using anti-Flag antibody M2 (#F-3165, Sigma-Aldrich, Saint Louis, USA) and goat anti-mouse-IgG1 IRDye 800CW antibody (Licor, Lincoln, USA).

### Purification of antibody variants by affinity chromatography

To purify Flag-tagged antibody variants, affinity chromatography with anti-Flag M2 agarose and Flag® peptide (both Sigma-Aldrich, Steinheim, Germany) was performed as described by the manufacturer. After elution, purity of the proteins was analyzed by SDS-PAGE and silver staining with the Pierce Silver Stain Kit (Thermo Fisher Scientific, MA, USA) according to the protocol of the manufacturer. The concentrations of the purified proteins were estimated by comparison with the proteins of known concentration of the Amersham LMW Calibration Kit for SDS Electrophoresis (GE Healthcare UK Limited, Little Chalfont, UK).

### High-performance liquid chromatography (HPLC)

The purified anti-CD40 fusion proteins were further analyzed regarding potential protein aggregation and degradation by size exclusion chromatography (SEC) by the UltiMate 3000 HPLC System (Thermo Fisher) with a MabPac SEC-1 column (#088460, Thermo Fisher). Calibration of the column was carried out with the aqueous SEC-1 column performance check standard (#AL0–3042, Phenomenex, Torrance, CA, USA).

### Coculture assays and IL8 ELISA

CD40-responsive cells (HT1080-CD40 or U2OS) were seeded in 96-well plates (2 × 10^4^ cells per well) and grown overnight. The next day, medium was changed to minimize the background of constitutive IL8 production, and cells were stimulated overnight with the anti-CD40 constructs as indicated. For cocultures CD40-responsive cells were supplemented with a similar number of HEK293 cells transfected with empty vector or expression vector encoding memCD40L or FcγRIIB along with the different antibody fusion proteins. The amount of IL8 in the supernatant was determined using the BD OptEIA^TM^ human IL8-ELISA kit (BD Biosciences, NJ, USA) according to the manufacturer’s protocol. Cocultures with memCD40L expressing transfectants served to define the maximal CD40-induced IL8 response.

### Western blot

To evaluate CD40-mediated activation we analyzed p100 processing and TRAF1 induction in U2OS cells and iDCs by western blotting. For western blot, coculture experiments were performed in six well plates (10^6^ +10^6^ cells per well). For stimulation of iDCs 0.8 × 10^6^ cells per well were seeded also in 6-well plates and stimulated overnight. Cells were collected in ice-cold PBS by scraping with a rubber policeman. Cells were then washed twice with fresh ice-cold PBS, pelleted (5 min, 4°C, 4630 g) and resuspended in Laemmli buffer. Samples were sonicated for 25 seconds at 100% amplitude with a sonication probe (UP100H Ultrasonic Processor, Helscher, Germany), heated at 95°C for 5 min and subjected to SDS-PAGE separation. After transfer of proteins to a nitrocellulose membrane western blot analysis was performed with an anti-p100/p52 (#05–361, Millipore), anti-TRAF1 (#4715), anti-A20 (#5630, both Cell Signaling Technology Beverly, MA, USA), anti-β-actin (#A1978–200), anti-Flag (M2) (#F-3165, both Sigma Aldrich) and horseradish peroxidase (HRP)-conjugated polyclonal rabbit anti-mouse antibody (#P0260, Dako, Glostrup, Denmark) or HRP-coupled anti-rabbit antibody (#7074, Cell Signaling Technology Beverly, MA, USA). Finally, membranes were developed by chemiluminescence western blot detection using ECL solution.

### Binding studies

For cellular binding studies with adherent cells, 2 × 10^4^ cells/well were cultured overnight in flat clear bottom black cell culture plates (Greiner Bio-One). To analyze the inhibitory effect of anti-CD40 antibodies on CD40L binding, HT1080-CD40 (and HT1080 to measure unspecific binding) cells were incubated with the anti-CD40 antibodies at 37°C for 30 minutes in triplicates and then supplemented with GpL-TNC-CD40L (100 ng/ml), a GpL fusion protein of soluble trimeric CD40L which has been stabilized by introducing the tenascin-c trimerization domain [49,52]. After one additional hour, cells were washed five times with ice-cold PBS, and cell-associated luciferase activity was measured.

For binding studies with HEK293 cells, they were transiently transfected with expression plasmid encoding FcγRIIB or empty vector (unspecific). Next day, cells were harvested, and aliquots were pairwise incubated with anti-CD40 (G28.5)-GpL-variants. Unbound anti-CD40-GpL constructs were removed by three washes with ice-cold PBS (centrifugation 1 min, 4°C, 21 300 g). Pellets were then resuspended in 50 µl of RPMI 1640 medium with 0.5% FCS and transferred to a black 96 well plate and GpL activity was measured. Specific binding was calculated by subtraction of the unspecific binding values from the corresponding total binding values.

To determine the specific binding of GpL-linked anti-CD40 antibodies, the latter were added pairwise with increasing concentrations to HT1080 (unspecific binding) and HT1080-CD40 (total binding) cells. After 1 hour at 37°C, cells were washed five times with fresh ice-cold PBS, and finally GpL activity was measured. To calculate specific binding the unspecific binding values were subtracted from the corresponding total binding values.

To analyze the binding of GpL-tagged deletion mutants of the ectodomain of CD40 to the various anti-CD40 antibodies, black high binding 96-well plates (Greiner Bio-One) were coated with 1 µg/ml protein G overnight. After blocking remaining free binding sites with 10% FCS in PBS and three washing steps with PBST, anti-CD40 antibodies (1 µg/ml) were added for 30 minutes in medium. After removal of free antibodies by three washing steps with PBST the GpL-linked CD40 deletion mutants were added for 1 hour at 37°C. Cells were then washed five times with ice-cold PBS, and luminescence was determined. GpL activities were measured with Lumo luminometer (anthos Mikrosysteme GmbH, Friesoythe, Germany) by adding 25 µl per well of 1.5 µM coelenterazine substrate (Carl Roth, Karlsruhe, Germany) in PBS.

### Generation and use of iDcs

Blood buffy coats of anonymous donors were obtained from the Institute of Clinical Transfusion Medicine and Hemotherapy of the University Hospital Würzburg. Peripheral blood mononuclear cells were isolated from blood buffy coats using density gradient centrifugation with lymphocyte separation medium (Histopaque 1077, Sigma-Aldrich, Germany). Pure monocytes were isolated using anti-CD14-coated beads and magnetic bead separation using LS columns (Miltenyi Biotec, Bergisch Gladbach, Germany). To obtain iDCs, monocytes were cultivated in 10 cm Petri dishes containing RPMI 1640, 10% FCS to induce the differentiation of monocytes into iDCs by adding 30 ng/ml of IL4 (Miltenyi Biotec) and 50 ng/ml of GM-CSF (Miltenyi Biotec) every third day for 6 days. Differentiation to iDCs was controlled by FACS evaluation for the absence of CD14 expression.

### Flow cytometry

To analyze maturation and activation of DCs, cells were tested by flow cytometry for the cell surface expression of CD14 and CD83. Cells were harvested and washed with PBS. Next, 2 × 10^6^ cells were resuspended in 100 µl PBS and incubated for 1 hour with the PE-labeled (CD14, CD83 from Miltenyi Biotec) antibodies or appropriate PE-labeled isotype controls (IgG1, IgG2a from R&D Systems, MN, USA) on ice. Following wash steps with PBS served to remove unbound antibodies. Finally, DCs were analyzed with an Attune NxT Flow Cytometer (Invitrogen, CA, USA).

## Supplementary Material

Supplemental data 222391703 revised.pdf

## Data Availability

The data that support the findings of this study are available from the corresponding author upon reasonable request or are already available within the article or its supplementary materials.
