# Supplementary material for: Genetically engineered IgG1 and nanobody oligomers acquire strong intrinsic CD40 agonism
Source: Bioengineered. 2024 Jan 12;15(1):2302246. doi: 10.1080/21655979.2024.2302246 (PMC10793706; doi:10.1080/21655979.2024.2302246)
Supplement: Supplemental data 222391703 revised.pdf [file KBIE_A_2302246_SM9904.pdf]

Supplemental Data

anti-CD40 antibody constructs with high intrinsic agonism

Nienke Hesen[1], Mohamed Anany[1], Andre Freidel[1], Mediya Baker[1], Daniela Siegmund[1], Olena Zaitseva[1], Harald Wajant[1,2] and Isabell Lang[1]

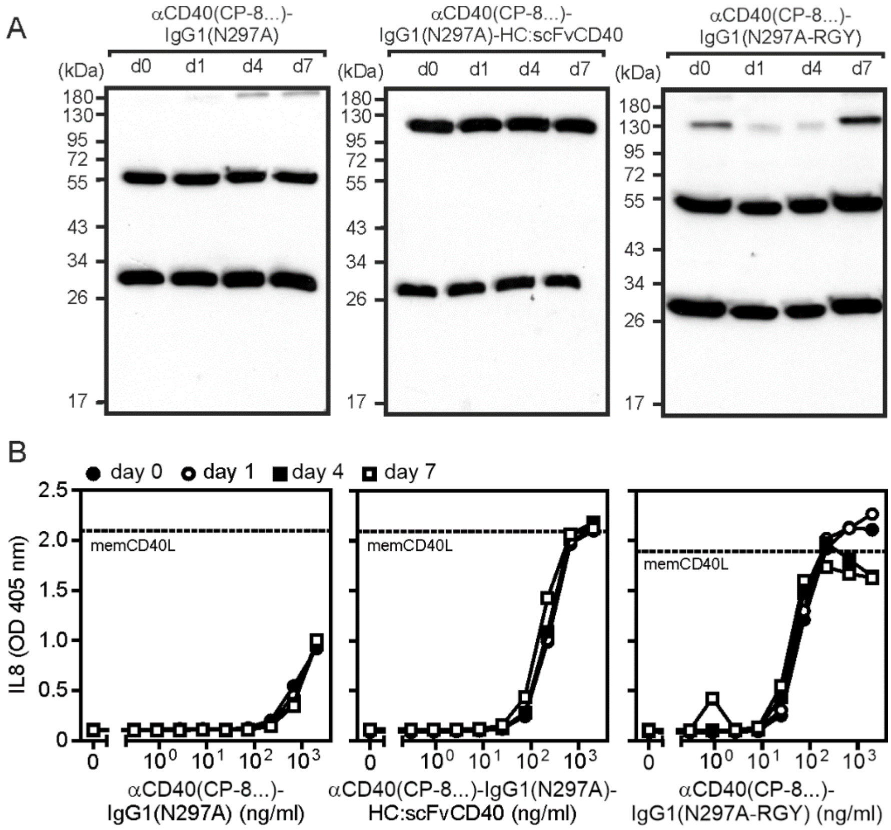

Supplemental Figure 1. Anti-CD40(CP-8…)-IgG1(N297A)-HC:scFvCD40 and anti-CD40(CP-8…)-IgG1(N297A-RGY) are stable at 37°C. (**A,B**) Anti-CD40(CP-8…)-IgG1(N297A)-HC:scFvCD40 and anti-CD40(CP-8…)-IgG1(N297A-RGY) along with their parental IgG1(N297A) variant were incubated for 1, 4 or 7 days at 37°C in medium supplemented with 2 % FCS. The samples were then analyzed by western blotting with anti-Flag mAb M2 (A) for integrity (A) and for agonistic activity by evaluation of IL8 induction in HT1080-CD40 cells (B). In the latter case, IL8 production of 1:1 cocultures with memCD40L transfected HEK293 cells served as benchmark.

Alt text: The figure shows western blot and activity data of samples of different variants of the anti-CD40 antibody CP-870,893 which have been incubated up to 7 days at 37°C. It is evident that this treatment did not affected integrity and activity of the constructs.

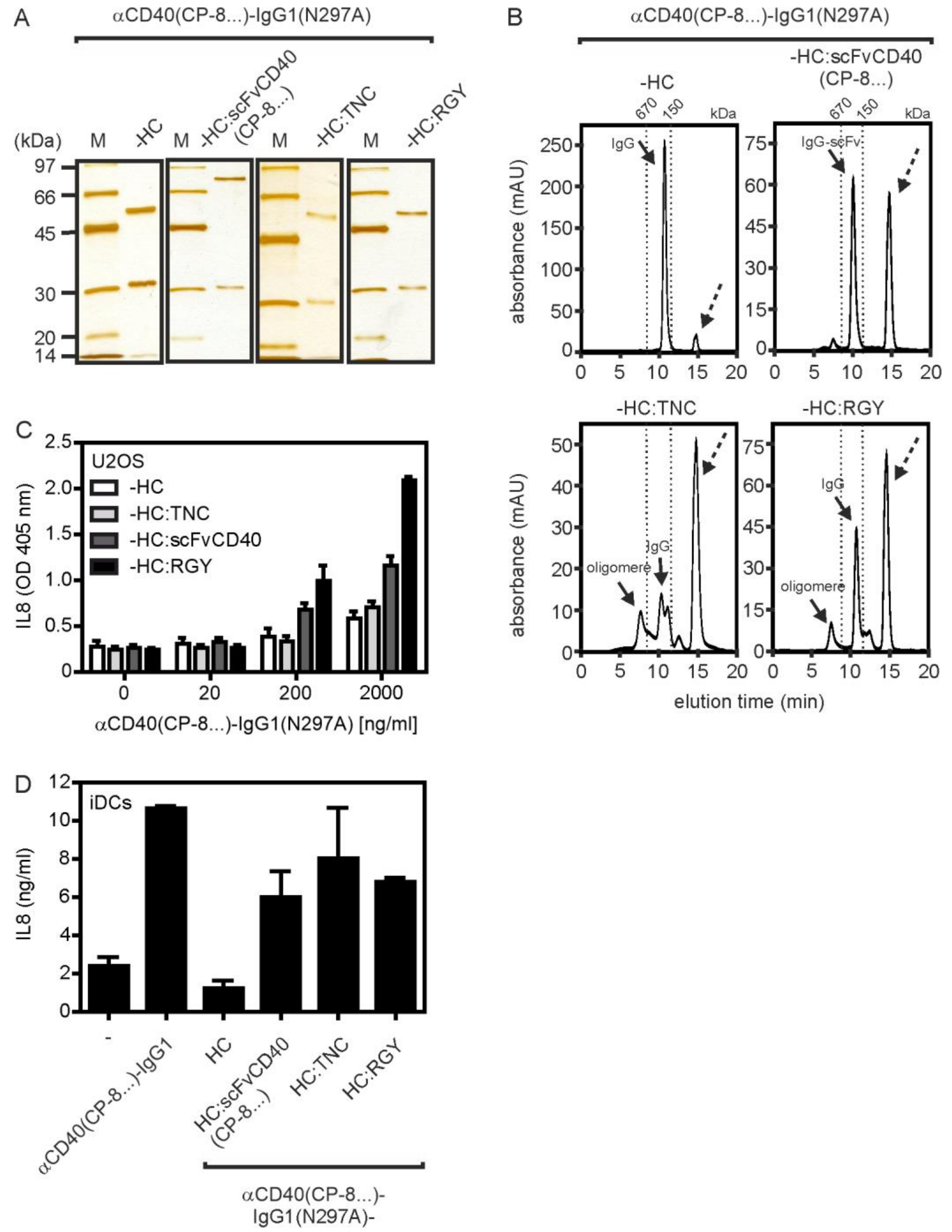

Supplemental Figure 2. Agonism of oligovalent anti-CD40(CP-8…)-IgG1(N297A) variants and anti-CD40(CP-8…)-IgG1(N297A)-HC:scFvCD40 fusion proteins is aggregation-independent. (**A**) The indicated constructs were purified by affinity chromatography on anti-Flag agarose and purity was evaluated by SDS-PAGE and silver staining. (**B**) Gel filtration analysis of the purified antibody fusion proteins. Dotted arrows indicate Flag peptide remained from the affinity purification. (**C**) IL8 induction in U2OS by purified anti-CD40(CP-8…)-IgG1(N297A) antibody variants. (**D**) Immature monocyte-derived dendritic cells (iDCs) were generated by cultivation of monocytes for 7 days with GM-CSF/IL4.

iDCs were then treated with 500 ng/ml of the indicated variants of anti-CD40(CP-8…). Next day, cell culture supernatants were analyzed by IL8 ELISA.

Alt text: The figure summarizes data showing purification and gel filtration data of oligomerized versions of the anti-CD40 antibody anti-CD40(CP-8…) along with functional data. It is evident that all oligomerized constructs are highly active.

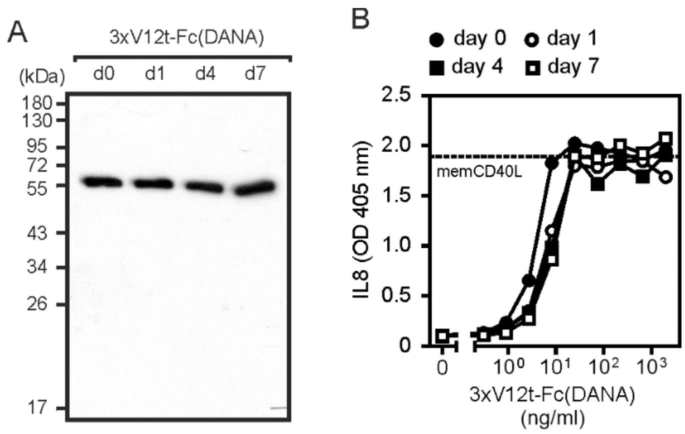

Supplemental Figure 3. 3xV12t-Fc(DANA) is stable at 37°C. (**A,B**) 3xV12t-Fc(DANA) was incubated for 1, 4 or 7 days at 37°C in medium supplemented with 2 % FCS. The samples were then analyzed by western blotting with anti-Flag mAb M2 (A) for integrity (A) and for agonistic activity by evaluation of IL8 induction in HT1080-CD40 cells (B). In the latter case, IL8 production of 1:1 cocultures with memCD40L transfected HEK293 cells served as benchmark.

Alt text: The figure shows western blot and activity data of a nonameric fusion protein (3xV12t-TNC) of the CD40-specific nanobody V12t which has been incubated up to 7 days at 37°C. It is evident that this treatment did not affected integrity and activity of the construct.

construct	peak elution time (min)	symbol
G28.5 (N297A-scFv)	9.3	—▲—
Apexi (N297A-scFv)	9.3	—●—
Seli (N297A-scFv)	9.4	—▼—
3xV12t-TNC	11	—×—

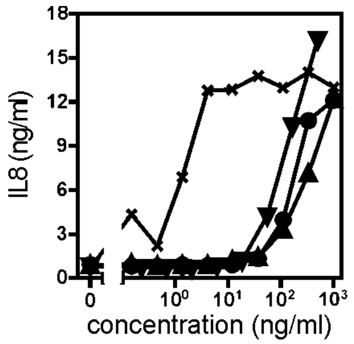

Supplemental Figure 4. CD40-stimulating activity of the correctly assembled molecule species of the IgG1(N297A)-HC:scFv variants of G28.5, APX005M (Apexi) and CP-870,893 (Seli), and the nonameric nanobody construct 3xV12t-TNC. The indicated proteins were initially purified by affinity chromatography using anti-Flag agarose and competitive elution with Flag peptide (100 µg/ml) in PBS as buffer. Purified proteins were subsequently subjected to gel filtration on a MabPac SEC-1 column (#088460, Thermo Fisher). Fractions of the peaks of corresponding to the size of the correctly assembled proteins were finally assayed on HT1080-CD40 cells for their IL8-inducing capacity.

Alt text: The figure shows IL8-induction by CD40 agonists purified by anti-Flag affinity purification and gel filtration.

Supplemental Table S1. pCR3-based expression plasmids and corresponding amino acid sequences.

Leader: underlined; Flag tag: underlined + grey background; restriction site encoding 2AA linker: bold; linker: bold + italic; variable domains: italic; TNC trimerization domain: italic + underlined + grey background; IgG1(N297A), IgG1(A+RGY), Fc, Fc(DANA), FAB1(1-108): grey background.

1	anti-CD40(G28.5)-Flag-VH-N297A-pCR3	MNFGFSLIFLVLVLKGVQCEVKLVPR**QL**DYKDDDDK**EL**DIQLQQSGPGLVKPSQSLSLT<br>CSVTGYSITTNYNWNWIRQFPGNKLEWMGYIRYDGTSEYTPSLKNRVSITRDTSMNQFF<br>LRLTSVTPEDTATYYCARLDYWGQGTLVTVSS**GS**SSASTKGPSVFPLAPSSKSTSGGTA<br>ALGCLVKDYFPEPVTVSWNSGALTSGVHTFPAVLQSSGLYSLSSVVTVPSSSLGTQTYI<br>CNVNHKPSNTKVDKKVEPKSCDKTHTCPPCPAPELLGGPSVFLFPPKPKDTLMISRTPE<br>VTCVVVDVSHEDPEVKFNWYVDGVEVHNAKTKPREEQYASTYRVVSVLTVLHQDWLNGK<br>EYKCKVSNKALPAPIEKTISKAKGQPREPQVYTLPPSRDELTKNQVSLTCLVKGFYPSD<br>IAVEWESNGQPENNYKTTPPVLDSDGSFFLYSKLTVDKSRWQQGNVFSCSVMHEALHNH<br>YTQKSLSLSPGK
2	anti-CD40(G28.5)-Flag-VH-IgG1-pCR3	MNFGFSLIFLVLVLKGVQCEVKLVPR**QL**DYKDDDDK**EL**DIQLQQSGPGLVKPSQSLSLT<br>CSVTGYSITTNYNWNWIRQFPGNKLEWMGYIRYDGTSEYTPSLKNRVSITRDTSMNQFF<br>LRLTSVTPEDTATYYCARLDYWGQGTLVTVSS**GS**SSASTKGPSVFPLAPSSKSTSGGTA<br>ALGCLVKDYFPEPVTVSWNSGALTSGVHTFPAVLQSSGLYSLSSVVTVPSSSLGTQTYI<br>CNVNHKPSNTKVDKKVEPKSCDKTHTCPPCPAPELLGGPSVFLFPPKPKDTLMISRTPE<br>VTCVVVDVSHEDPEVKFNWYVDGVEVHNAKTKPREEQYNSTYRVVSVLTVLHQDWLNGK<br>EYKCKVSNKALPAPIEKTISKAKGQPREPQVYTLPPSRDELTKNQVSLTCLVKGFYPSD<br>IAVEWESNGQPENNYKTTPPVLDSDGSFFLYSKLTVDKSRWQQGNVFSCSVMHEALHNH<br>YTQKSLSLSPGK
3	anti-CD40(G28.5)-Flag-VH-IgG2-pCR3	MNFGFSLIFLVLVLKGVQCEVKLVPR**QL**DYKDDDDK**EL**DIQLQQSGPGLVKPSQSLSLT<br>CSVTGYSITTNYNWNWIRQFPGNKLEWMGYIRYDGTSEYTPSLKNRVSITRDTSMNQFF<br>LRLTSVTPEDTATYYCARLDYWGQGTLVTVSS**GS**SSASTKGPSVFPLAPCSRSTSESTA<br>ALGCLVKDYFPEPVTVSWNSGALTSGVHTFPAVLQSSGLYSLSSVVTVPSSNFGTQTYT<br>CNVDHKPSNTKVDKTVERKCCVECPPCPAPPVAGPSVFLFPPKPKDTLMISRTPEVTCV<br>VVDVSHEDPEVQFNWYVDGVEVHNAKTKPREEQFNSTFRVVSVLTVVHQDWLNGKEYKC<br>KVSNKGLPAPIEKTISKTKGQPREPQVYTLPPSREEMTKNQVSLTCLVKGFYPSDIAVE<br>WESNGQPENNYKTTPPMLDSDGSFFLYSKLTVDKSRWQQGNVFSCSVMHEALHNHYTQK<br>SLSLSPGK
4	anti-CD40(G28.5)-Flag-VH-IgG4-pCR3	MNFGFSLIFLVLVLKGVQCEVKLVPR**QL**DYKDDDDK**EL**DIQLQQSGPGLVKPSQSLSLT<br>CSVTGYSITTNYNWNWIRQFPGNKLEWMGYIRYDGTSEYTPSLKNRVSITRDTSMNQFF<br>LRLTSVTPEDTATYYCARLDYWGQGTLVTVSS**GS**SSASTKGPSVFPLAPCSRSTSESTA<br>ALGCLVKDYFPEPVTVSWNSGALTSGVHTFPAVLQSSGLYSLSSVVTVPSSSLGTKTYT<br>CNVDHKPSNTKVDKRVESKYGPPCPSCPAPEFLGGPSVFLFPPKPKDTLMISRTPEVTC<br>VVVDVSQEDPEVQFNWYVDGVEVHNAKTKPREEQFNSTYRVVSVLTVLHQDWLNGKEYK<br>CKVSNKGLPSSIEKTISKAKGQPREPQVYTLPPSQEEMTKNQVSLTCLVKGFYPSDIAV<br>EWESNGQPENNYKTTPPVLDSDGSFFLYSRLTVDKSRWQEGNVFSCSVMHEALHNHYTQ<br>KSLSLSPGK
5	anti-CD40(G28.5)-Flag-VH-N297A-TNC-pCR3	MNFGFSLIFLVLVLKGVQCEVKLVPR**QL**DYKDDDDK**EL**DIQLQQSGPGLVKPSQSLSLT<br>CSVTGYSITTNYNWNWIRQFPGNKLEWMGYIRYDGTSEYTPSLKNRVSITRDTSMNQFF<br>LRLTSVTPEDTATYYCARLDYWGQGTLVTVSS**GS**SSASTKGPSVFPLAPSSKSTSGGTA<br>ALGCLVKDYFPEPVTVSWNSGALTSGVHTFPAVLQSSGLYSLSSVVTVPSSSLGTQTYI<br>CNVNHKPSNTKVDKKVEPKSCDKTHTCPPCPAPELLGGPSVFLFPPKPKDTLMISRTPE<br>VTCVVVDVSHEDPEVKFNWYVDGVEVHNAKTKPREEQYASTYRVVSVLTVLHQDWLNGK<br>EYKCKVSNKALPAPIEKTISKAKGQPREPQVYTLPPSRDELTKNQVSLTCLVKGFYPSD<br>IAVEWESNGQPENNYKTTPPVLDSDGSFFLYSKLTVDKSRWQQGNVFSCSVMHEALHNH<br>YTQKSLSLSPGK**LE**DIACGCAAAPDIKDLLSRLEELEGLVSSLREQGTG
6	anti-CD40(G28.5)-Flag-VH-IgG1(N297A+E374R/E459G/S469Y)-pCR3	MNFGFSLIFLVLVLKGVQCEVKLVPR**QL**DYKDDDDK**EL**DIQLQQSGPGLVKPSQSLSLT<br>CSVTGYSITTNYNWNWIRQFPGNKLEWMGYIRYDGTSEYTPSLKNRVSITRDTSMNQFF<br>LRLTSVTPEDTATYYCARLDYWGQGTLVTVSS**GS**SSASTKGPSVFPLAPSSKSTSGGTA<br>ALGCLVKDYFPEPVTVSWNSGALTSGVHTFPAVLQSSGLYSLSSVVTVPSSSLGTQTYI<br>CNVNHKPSNTKVDKKVEPKSCDKTHTCPPCPAPELLGGPSVFLFPPKPKDTLMISRTPE<br>VTCVVVDVSHEDPEVKFNWYVDGVEVHNAKTKPREEQYASTYRVVSVLTVLHQDWLNGK<br>EYKCKVSNKALPAPIEKTISKAKGQPRRPQVYTLPPSRDELTKNQVSLTCLVKGFYPSD<br>IAVEWESNGQPENNYKTTPPVLDSDGSFFLYSKLTVDKSRWQQGNVFSCSVMHGALHNH<br>YTQKYLSLSPGK
7	anti-CD40(G28.5)-Flag-VH-N297A-scFv-anti-CD40(G28.5)-pCR3	MNFGFSLIFLVLVLKGVQCEVKLVPR**QL**DYKDDDDK**EL**DIQLQQSGPGLVKPSQSLSLT<br>CSVTGYSITTNYNWNWIRQFPGNKLEWMGYIRYDGTSEYTPSLKNRVSITRDTSMNQFF<br>LRLTSVTPEDTATYYCARLDYWGQGTLVTVSS**GS**SSASTKGPSVFPLAPSSKSTSGGTA<br>ALGCLVKDYFPEPVTVSWNSGALTSGVHTFPAVLQSSGLYSLSSVVTVPSSSLGTQTYI<br>CNVNHKPSNTKVDKKVEPKSCDKTHTCPPCPAPELLGGPSVFLFPPKPKDTLMISRTPE<br>VTCVVVDVSHEDPEVKFNWYVDGVEVHNAKTKPREEQYASTYRVVSVLTVLHQDWLNGK<br>EYKCKVSNKALPAPIEKTISKAKGQPREPQVYTLPPSRDELTKNQVSLTCLVKGFYPSD<br>IAVEWESNGQPENNYKTTPPVLDSDGSFFLYSKLTVDKSRWQQGNVFSCSVMHEALHNH<br>YTQKSLSLSPGK**LE**DIVMTQNPLSLPVSLGDEASISCRSSQSLENSNGNTFLNWFFQKP<br>GQSPQLLIYRVSNRFSGVPDRFSGSGSGTDFTLKISRVEAEDLGVYFCLQVTHVPYTFG<br>GGTTLEIK***GGGGSGGGGSGGGGS***DIQLQQSGPGLVKPSQSLSLTCSVTGYSITTNYNWN

		WIRQFPGNKLEWMGYIRYDGTSEYTPSLKNRVSITRDTSMNQFFLRLTSVTPEDTATYY<br>CARLDYWGQGTLVTVSS
8	anti-CD40(G28.5)-Flag-VL-light-pCR3	MNFGFSLIFLVLVLKGVQCEVKLVPR**QL**DYKDDDDK**EL**DIVMTQNPLSLPVSLGDEASI<br>SCRSSQSLENSNGNTFLNWFFQKPGQSPQLLIYRVSNRFSGVPDRFSGSGSGTDFTLKI<br>SRVEAEDLGVYFCLQVTHVPYTFGGGTTLEIK**GS**EIKRTVAAPSVFIFPPSDEQLKSGT<br>ASVVCLLNNFYPREAKVQWKVDNALQSGNSQESVTEQDSKDSTYSLSSTLTLSKADYEK<br>HKVYACEVTHQGLSSPVTKSFNRGEC
9	anti-CD40(ChiLob)-Flag-VH-IgG1(N297A+ E374R/E459G/S469Y)-pCR3	MNFGFSLIFLVLVLKGVQCEVKLVPR**QL**DYKDDDDK**EF**EVQLQQSGPDLVKPGASVKIS<br>CKTSGYTFTEYIMHWVKQSHGKSLEWIGGIIPNNGGTSYNQKFKDKATMTVDKSSSTGY<br>MELRSLTSEDSAVYYCTRREVYGRNYYALDYWGQGTLVTVSS**RS**SSASTKGPSVFPLAP<br>SSKSTSGGTAALGCLVKDYFPEPVTVSWNSGALTSGVHTFPAVLQSSGLYSLSSVVTVP<br>SSSLGTQTYICNVNHKPSNTKVDKKVEPKSCDKTHTCPPCPAPELLGGPSVFLFPPKPK<br>DTLMISRTPEVTCVVVDVSHEDPEVKFNWYVDGVEVHNAKTKPREEQYASTYRVVSVLT<br>VLHQDWLNGKEYKCKVSNKALPAPIEKTISKAKGQPRRPQVYTLPPSRDELTKNQVSLT<br>CLVKGFYPSDIAVEWESNGQPENNYKTTPPVLDSDGSFFLYSKLTVDKSRWQQGNVFSC<br>SVMHGALHNHYTQKYLSLSPGK
10	anti-CD40(ChiLob)-Flag-VH-N297A-pCR3	MNFGFSLIFLVLVLKGVQCEVKLVPR**QL**DYKDDDDK**EF**EVQLQQSGPDLVKPGASVKIS<br>CKTSGYTFTEYIMHWVKQSHGKSLEWIGGIIPNNGGTSYNQKFKDKATMTVDKSSSTGY<br>MELRSLTSEDSAVYYCTRREVYGRNYYALDYWGQGTLVTVSS**RS**SSASTKGPSVFPLAP<br>SSKSTSGGTAALGCLVKDYFPEPVTVSWNSGALTSGVHTFPAVLQSSGLYSLSSVVTVP<br>SSSLGTQTYICNVNHKPSNTKVDKKVEPKSCDKTHTCPPCPAPELLGGPSVFLFPPKPK<br>DTLMISRTPEVTCVVVDVSHEDPEVKFNWYVDGVEVHNAKTKPREEQYASTYRVVSVLT<br>VLHQDWLNGKEYKCKVSNKALPAPIEKTISKAKGQPREPQVYTLPPSRDELTKNQVSLT<br>CLVKGFYPSDIAVEWESNGQPENNYKTTPPVLDSDGSFFLYSKLTVDKSRWQQGNVFSC<br>SVMHEALHNHYTQKSLSLSPGK
11	anti-CD40(ChiLob)-Flag-VH-N297A-scFv-anti-CD40(ChiLob)-pCR3	MNFGFSLIFLVLVLKGVQCEVKLVPR**QL**DYKDDDDK**EF**EVQLQQSGPDLVKPGASVKIS<br>CKTSGYTFTEYIMHWVKQSHGKSLEWIGGIIPNNGGTSYNQKFKDKATMTVDKSSSTGY<br>MELRSLTSEDSAVYYCTRREVYGRNYYALDYWGQGTLVTVSS**RS**SSASTKGPSVFPLAP<br>SSKSTSGGTAALGCLVKDYFPEPVTVSWNSGALTSGVHTFPAVLQSSGLYSLSSVVTVP<br>SSSLGTQTYICNVNHKPSNTKVDKKVEPKSCDKTHTCPPCPAPELLGGPSVFLFPPKPK<br>DTLMISRTPEVTCVVVDVSHEDPEVKFNWYVDGVEVHNAKTKPREEQYASTYRVVSVLT<br>VLHQDWLNGKEYKCKVSNKALPAPIEKTISKAKGQPREPQVYTLPPSRDELTKNQVSLT<br>CLVKGFYPSDIAVEWESNGQPENNYKTTPPVLDSDGSFFLYSKLTVDKSRWQQGNVFSC<br>SVMHEALHNHYTQKSLSLSPGK**LE**EVQLQQSGPDLVKPGASVKISCKTSGYTFTEYIMH<br>WVKQSHGKSLEWIGGIIPNNGGTSYNQKFKDKATMTVDKSSSTGYMELRSLTSEDSAVY<br>YCTRREVYGRNYYALDYWGQGTLVTVSS**RSSTKGPKLEEGEFSEAQL**DIQMTQTTSSLS<br>ASLGDRVTITCSASQGINNYLNWYQQKPDGTVKLLIYYTSSLHSGVPSRFSGSGSGTDY<br>SLTISNLEPEDIATYYCQQYSNLPYTFGGGTKLEIK
12	anti-CD40(ChiLob)-Flag-VH-N297A-TNC-pCR3	MNFGFSLIFLVLVLKGVQCEVKLVPR**QL**DYKDDDDK**EF**EVQLQQSGPDLVKPGASVKIS<br>CKTSGYTFTEYIMHWVKQSHGKSLEWIGGIIPNNGGTSYNQKFKDKATMTVDKSSSTGY<br>MELRSLTSEDSAVYYCTRREVYGRNYYALDYWGQGTLVTVSS**RS**SSASTKGPSVFPLAP<br>SSKSTSGGTAALGCLVKDYFPEPVTVSWNSGALTSGVHTFPAVLQSSGLYSLSSVVTVP<br>SSSLGTQTYICNVNHKPSNTKVDKKVEPKSCDKTHTCPPCPAPELLGGPSVFLFPPKPK<br>DTLMISRTPEVTCVVVDVSHEDPEVKFNWYVDGVEVHNAKTKPREEQYASTYRVVSVLT<br>VLHQDWLNGKEYKCKVSNKALPAPIEKTISKAKGQPREPQVYTLPPSRDELTKNQVSLT<br>CLVKGFYPSDIAVEWESNGQPENNYKTTPPVLDSDGSFFLYSKLTVDKSRWQQGNVFSC<br>SVMHEALHNHYTQKSLSLSPGK**LE**DIACGCAAAPDIKDLLSRLEELEGLVSSLREQGTG
13	anti-CD40(ChiLob)-Flag-VH-IgG1-pCR3	MNFGFSLIFLVLVLKGVQCEVKLVPR**QL**DYKDDDDK**EF**EVQLQQSGPDLVKPGASVKIS<br>CKTSGYTFTEYIMHWVKQSHGKSLEWIGGIIPNNGGTSYNQKFKDKATMTVDKSSSTGY<br>MELRSLTSEDSAVYYCTRREVYGRNYYALDYWGQGTLVTVSS**RS**SSASTKGPSVFPLAP<br>SSKSTSGGTAALGCLVKDYFPEPVTVSWNSGALTSGVHTFPAVLQSSGLYSLSSVVTVP<br>SSSLGTQTYICNVNHKPSNTKVDKKVEPKSCDKTHTCPPCPAPELLGGPSVFLFPPKPK<br>DTLMISRTPEVTCVVVDVSHEDPEVKFNWYVDGVEVHNAKTKPREEQYNSTYRVVSVLT<br>VLHQDWLNGKEYKCKVSNKALPAPIEKTISKAKGQPREPQVYTLPPSRDELTKNQVSLT<br>CLVKGFYPSDIAVEWESNGQPENNYKTTPPVLDSDGSFFLYSKLTVDKSRWQQGNVFSC<br>SVMHEALHNHYTQKSLSLSPGK
14	anti-CD40(ChiLob)-Flag-VH-IgG2-pCR3	MNFGFSLIFLVLVLKGVQCEVKLVPR**QL**DYKDDDDK**EF**EVQLQQSGPDLVKPGASVKIS<br>CKTSGYTFTEYIMHWVKQSHGKSLEWIGGIIPNNGGTSYNQKFKDKATMTVDKSSSTGY<br>MELRSLTSEDSAVYYCTRREVYGRNYYALDYWGQGTLVTVSS**RS**SSASTKGPSVFPLAP<br>CSRSTSESTAALGCLVKDYFPEPVTVSWNSGALTSGVHTFPAVLQSSGLYSLSSVVTVP<br>SSNFGTQTYTCNVDHKPSNTKVDKTVERKCCVECPPCPAPPVAGPSVFLFPPKPKDTLM<br>ISRTPEVTCVVVDVSHEDPEVQFNWYVDGVEVHNAKTKPREEQFNSTFRVVSVLTVVHQ<br>DWLNGKEYKCKVSNKGLPAPIEKTISKTKGQPREPQVYTLPPSREEMTKNQVSLTCLVK<br>GFYPSDIAVEWESNGQPENNYKTTPPMLDSDGSFFLYSKLTVDKSRWQQGNVFSCSVMH<br>EALHNHYTQKSLSLSPGK
15	anti-CD40(ChiLob)-Flag-VH-IgG4-pCR3	MNFGFSLIFLVLVLKGVQCEVKLVPR**QL**DYKDDDDK**EF**EVQLQQSGPDLVKPGASVKIS<br>CKTSGYTFTEYIMHWVKQSHGKSLEWIGGIIPNNGGTSYNQKFKDKATMTVDKSSSTGY<br>MELRSLTSEDSAVYYCTRREVYGRNYYALDYWGQGTLVTVSS**RS**SSASTKGPSVFPLAP<br>CSRSTSESTAALGCLVKDYFPEPVTVSWNSGALTSGVHTFPAVLQSSGLYSLSSVVTVP<br>SSSLGTKTYTCNVDHKPSNTKVDKRVESKYGPPCPSCPAPEFLGGPSVFLFPPKPKDTL<br>MISRTPEVTCVVVDVSQEDPEVQFNWYVDGVEVHNAKTKPREEQFNSTYRVVSVLTVLH<br>QDWLNGKEYKCKVSNKGLPSSIEKTISKAKGQPREPQVYTLPPSQEEMTKNQVSLTCLV<br>KGFYPSDIAVEWESNGQPENNYKTTPPVLDSDGSFFLYSRLTVDKSRWQEGNVFSCSVM<br>HEALHNHYTQKSLSLSPGK
16	anti-CD40(ChiLob)-Flag-VL-light-pCR3	MNFGFSLIFLVLVLKGVQCEVKLVPR**QL**DYKDDDDK**EL**DIQMTQTTSSLSASLGDRVTI<br>TCSASQGINNYLNWYQQKPDGTVKLLIYYTSSLHSGVPSRFSGSGSGTDYSLTISNLEP<br>EDIATYYCQQYSNLPYTFGGGTKLEIK**GS**EIKRTVAAPSVFIFPPSDEQLKSGTASVVC

		LLNNFYPREAKVQWKVDNALQSGNSQESVTEQDSKDSTYSLSSTLTLSKADYEKHKVYA<br>CEVTHQGLSSPVTKSFNRGEC
17	anti-CD40(ADC)-Flag-VH-N297A-pCR3	MNFGFSLIFLVLVLKGVQCEVKLVPR**QL**DYKDDDDK**EF**EVQLLESGGGLVQPGGSLRLS<br>CAASGFTFSTYGMHWVRQAPGKGLEWLSYISGGSSYIFYADSVRGRFTISRDNSENALY<br>LQMNSLRAEDTAVYYCARILRGGSGMDLWGQGTLVTVSS**RS**SSASTKGPSVFPLAPSSK<br>STSGGTAALGCLVKDYFPEPVTVSWNSGALTSGVHTFPAVLQSSGLYSLSSVVTVPSSS<br>LGTQTYICNVNHKPSNTKVDKKVEPKSCDKTHTCPPCPAPELLGGPSVFLFPPKPKDTL<br>MISRTPEVTCVVVDVSHEDPEVKFNWYVDGVEVHNAKTKPREEQYASTYRVVSVLTVLH<br>QDWLNGKEYKCKVSNKALPAPIEKTISKAKGQPREPQVYTLPPSRDELTKNQVSLTCLV<br>KGFYPSDIAVEWESNGQPENNYKTTPPVLDSDGSFFLYSKLTVDKSRWQQGNVFSCSVM<br>HEALHNHYTQKSLSLSPGK
18	anti-CD40(ADC)-Flag-VH-IgG1(N297A+E374R/E459G/S469Y)-pCR3	MNFGFSLIFLVLVLKGVQCEVKLVPR**QL**DYKDDDDK**EF**EVQLLESGGGLVQPGGSLRLS<br>CAASGFTFSTYGMHWVRQAPGKGLEWLSYISGGSSYIFYADSVRGRFTISRDNSENALY<br>LQMNSLRAEDTAVYYCARILRGGSGMDLWGQGTLVTVSS**RS**SSASTKGPSVFPLAPSSK<br>STSGGTAALGCLVKDYFPEPVTVSWNSGALTSGVHTFPAVLQSSGLYSLSSVVTVPSSS<br>LGTQTYICNVNHKPSNTKVDKKVEPKSCDKTHTCPPCPAPELLGGPSVFLFPPKPKDTL<br>MISRTPEVTCVVVDVSHEDPEVKFNWYVDGVEVHNAKTKPREEQYASTYRVVSVLTVLH<br>QDWLNGKEYKCKVSNKALPAPIEKTISKAKGQPRRPQVYTLPPSRDELTKNQVSLTCLV<br>KGFYPSDIAVEWESNGQPENNYKTTPPVLDSDGSFFLYSKLTVDKSRWQQGNVFSCSVM<br>HGALHNHYTQKYLSLSPGK
19	anti-CD40(ADC)-Flag-VH-IgG1-pCR3	MNFGFSLIFLVLVLKGVQCEVKLVPR**QL**DYKDDDDK**EF**EVQLLESGGGLVQPGGSLRLS<br>CAASGFTFSTYGMHWVRQAPGKGLEWLSYISGGSSYIFYADSVRGRFTISRDNSENALY<br>LQMNSLRAEDTAVYYCARILRGGSGMDLWGQGTLVTVSS**RS**SSASTKGPSVFPLAPSSK<br>STSGGTAALGCLVKDYFPEPVTVSWNSGALTSGVHTFPAVLQSSGLYSLSSVVTVPSSS<br>LGTQTYICNVNHKPSNTKVDKKVEPKSCDKTHTCPPCPAPELLGGPSVFLFPPKPKDTL<br>MISRTPEVTCVVVDVSHEDPEVKFNWYVDGVEVHNAKTKPREEQYNSTYRVVSVLTVLH<br>QDWLNGKEYKCKVSNKALPAPIEKTISKAKGQPREPQVYTLPPSRDELTKNQVSLTCLV<br>KGFYPSDIAVEWESNGQPENNYKTTPPVLDSDGSFFLYSKLTVDKSRWQQGNVFSCSVM<br>HEALHNHYTQKSLSLSPGK
20	anti-CD40(ADC)-Flag-VH-IgG2-pCR3	MNFGFSLIFLVLVLKGVQCEVKLVPR**QL**DYKDDDDK**EF**EVQLLESGGGLVQPGGSLRLS<br>CAASGFTFSTYGMHWVRQAPGKGLEWLSYISGGSSYIFYADSVRGRFTISRDNSENALY<br>LQMNSLRAEDTAVYYCARILRGGSGMDLWGQGTLVTVSS**RS**SSASTKGPSVFPLAPCSR<br>STSESTAALGCLVKDYFPEPVTVSWNSGALTSGVHTFPAVLQSSGLYSLSSVVTVPSSN<br>FGTQTYTCNVDHKPSNTKVDKTVERKCCVECPPCPAPPVAGPSVFLFPPKPKDTLMISR<br>TPEVTCVVVDVSHEDPEVQFNWYVDGVEVHNAKTKPREEQFNSTFRVVSVLTVVHQDWL<br>NGKEYKCKVSNKGLPAPIEKTISKTKGQPREPQVYTLPPSREEMTKNQVSLTCLVKGFY<br>PSDIAVEWESNGQPENNYKTTPPMLDSDGSFFLYSKLTVDKSRWQQGNVFSCSVMHEAL<br>HNHYTQKSLSLSPGK
21	anti-CD40(ADC)-Flag-VH-IgG4-pCR3	MNFGFSLIFLVLVLKGVQCEVKLVPR**QL**DYKDDDDK**EF**EVQLLESGGGLVQPGGSLRLS<br>CAASGFTFSTYGMHWVRQAPGKGLEWLSYISGGSSYIFYADSVRGRFTISRDNSENALY<br>LQMNSLRAEDTAVYYCARILRGGSGMDLWGQGTLVTVSS**RS**SSASTKGPSVFPLAPCSR<br>STSESTAALGCLVKDYFPEPVTVSWNSGALTSGVHTFPAVLQSSGLYSLSSVVTVPSSS<br>LGTKTYTCNVDHKPSNTKVDKRVESKYGPPCPSCPAPEFLGGPSVFLFPPKPKDTLMIS<br>RTPEVTCVVVDVSQEDPEVQFNWYVDGVEVHNAKTKPREEQFNSTYRVVSVLTVLHQDW<br>LNGKEYKCKVSNKGLPSSIEKTISKAKGQPREPQVYTLPPSQEEMTKNQVSLTCLVKGF<br>YPSDIAVEWESNGQPENNYKTTPPVLDSDGSFFLYSRLTVDKSRWQEGNVFSCSVMHEA<br>LHNHYTQKSLSLSPGK
22	anti-CD40(ADC)-Flag-VH-N297A-scFv-anti-CD40(ADC)-pCR3	MNFGFSLIFLVLVLKGVQCEVKLVPR**QL**DYKDDDDK**EF**EVQLLESGGGLVQPGGSLRLS<br>CAASGFTFSTYGMHWVRQAPGKGLEWLSYISGGSSYIFYADSVRGRFTISRDNSENALY<br>LQMNSLRAEDTAVYYCARILRGGSGMDLWGQGTLVTVSS**RS**SSASTKGPSVFPLAPSSK<br>STSGGTAALGCLVKDYFPEPVTVSWNSGALTSGVHTFPAVLQSSGLYSLSSVVTVPSSS<br>LGTQTYICNVNHKPSNTKVDKKVEPKSCDKTHTCPPCPAPELLGGPSVFLFPPKPKDTL<br>MISRTPEVTCVVVDVSHEDPEVKFNWYVDGVEVHNAKTKPREEQYASTYRVVSVLTVLH<br>QDWLNGKEYKCKVSNKALPAPIEKTISKAKGQPREPQVYTLPPSRDELTKNQVSLTCLV<br>KGFYPSDIAVEWESNGQPENNYKTTPPVLDSDGSFFLYSKLTVDKSRWQQGNVFSCSVM<br>HEALHNHYTQKSLSLSPGK**LE**EVQLLESGGGLVQPGGSLRLSCAASGFTFSTYGMHWVR<br>QAPGKGLEWLSYISGGSSYIFYADSVRGRFTISRDNSENALYLQMNSLRAEDTAVYYCA<br>RILRGGSGMDLWGQGTLVTVSS**RSSTKGPKLEEGEFSEAQL**QSVLTQPPSASGTPGQRV<br>TISCTGSSSNIGAGYNVYWYQQLPGTAPKLLIYGNINRPSGVPDRFSGSKSGTSASLAI<br>SGLRSEDEADYYCAAWDKSISGLVFGGGTKLTVLG
23	anti-CD40(ADC)-Flag-VH-N297A-TNC-pCR3	MNFGFSLIFLVLVLKGVQCEVKLVPR**QL**DYKDDDDK**EF**EVQLLESGGGLVQPGGSLRLS<br>CAASGFTFSTYGMHWVRQAPGKGLEWLSYISGGSSYIFYADSVRGRFTISRDNSENALY<br>LQMNSLRAEDTAVYYCARILRGGSGMDLWGQGTLVTVSS**RS**SSASTKGPSVFPLAPSSK<br>STSGGTAALGCLVKDYFPEPVTVSWNSGALTSGVHTFPAVLQSSGLYSLSSVVTVPSSS<br>LGTQTYICNVNHKPSNTKVDKKVEPKSCDKTHTCPPCPAPELLGGPSVFLFPPKPKDTL<br>MISRTPEVTCVVVDVSHEDPEVKFNWYVDGVEVHNAKTKPREEQYASTYRVVSVLTVLH<br>QDWLNGKEYKCKVSNKALPAPIEKTISKAKGQPREPQVYTLPPSRDELTKNQVSLTCLV<br>KGFYPSDIAVEWESNGQPENNYKTTPPVLDSDGSFFLYSKLTVDKSRWQQGNVFSCSVM<br>HEALHNHYTQKSLSLSPGK**LE**DIACGCAAAPDIKDLLSRLEELEGLVSSLREQGTG
24	anti-CD40(ADC)-Flag-VL-light-pCR3	MNFGFSLIFLVLVLKGVQCEVKLVPR**QL**DYKDDDDK**EL**QSVLTQPPSASGTPGQRVTIS<br>CTGSSSNIGAGYNVYWYQQLPGTAPKLLIYGNINRPSGVPDRFSGSKSGTSASLAISGL<br>RSEDEADYYCAAWDKSISGLVFGGGTKLTVLG**GS**EIKRTVAAPSVFIFPPSDEQLKSGT<br>ASVVCLLNNFYPREAKVQWKVDNALQSGNSQESVTEQDSKDSTYSLSSTLTLSKADYEK<br>HKVYACEVTHQGLSSPVTKSFNRGEC
25	anti-CD40(APX)-Flag-VH-IgG1(N297A+	MNFGFSLIFLVLVLKGVQCEVKLVPR**QL**DYKDDDDK**EF**QSLEESGGDLVKPGASLTLTC<br>TASGFSFSSTYVCWVRQAPGKGLEWIACIYTGDGTNYSASWAKGRFTISKPSSTTVTLQ<br>MTSLTPADTATYFCARPDITYGFAINFWGPGTLVTVSS**RS**SSASTKGPSVFPLAPSSKS

	E374R/E459G/S469Y)-pCR3	TSGGTAALGCLVKDYFPEPVTVSWNSGALTSGVHTFPAVLQSSGLYSLSSVVTVPSSSL<br>GTQTYICNVNHKPSNTKVDKKVEPKSCDKTHTCPPCPAPELLGGPSVFLFPPKPKDTLM<br>ISRTPEVTCVVVDVSHEDPEVKFNWYVDGVEVHNAKTKPREEQYASTYRVVSVLTVLHQ<br>DWLNGKEYKCKVSNKALPAPIEKTISKAKGQPRRPQVYTLPPSRDELTKNQVSLTCLVK<br>GFYPSDIAVEWESNGQPENNYKTTPPVLDSDGSFFLYSKLTVDKSRWQQGNVFSCSVMH<br>GALHNHYTQKYLSLSPGK
26	anti-CD40(APX)-Flag-VH-IgG1-pCR3	MNFGFSLIFLVLVLKGVQCEVKLVPR**QL**DYKDDDDK**EF***QSLEESGGDLVKPGASLTLTC*<br>*TASGFSFSSTYVCWVRQAPGKGLEWIACIYTGDGTNYSASWAKGRFTISKPSSTTVTLQ*<br>*MTSLTPADTATYFCARPDITYGFAINFWGPGTLVTVSS***RS**SSASTKGPSVFPLAPSSKS<br>TSGGTAALGCLVKDYFPEPVTVSWNSGALTSGVHTFPAVLQSSGLYSLSSVVTVPSSSL<br>GTQTYICNVNHKPSNTKVDKKVEPKSCDKTHTCPPCPAPELLGGPSVFLFPPKPKDTLM<br>ISRTPEVTCVVVDVSHEDPEVKFNWYVDGVEVHNAKTKPREEQYNSTYRVVSVLTVLHQ<br>DWLNGKEYKCKVSNKALPAPIEKTISKAKGQPREPQVYTLPPSRDELTKNQVSLTCLVK<br>GFYPSDIAVEWESNGQPENNYKTTPPVLDSDGSFFLYSKLTVDKSRWQQGNVFSCSVMH<br>EALHNHYTQKSLSLSPGK
27	anti-CD40(APX)-Flag-VH-IgG2-pCR3	MNFGFSLIFLVLVLKGVQCEVKLVPR**QL**DYKDDDDK**EF***QSLEESGGDLVKPGASLTLTC*<br>*TASGFSFSSTYVCWVRQAPGKGLEWIACIYTGDGTNYSASWAKGRFTISKPSSTTVTLQ*<br>*MTSLTPADTATYFCARPDITYGFAINFWGPGTLVTVSS***RS**SSASTKGPSVFPLAPCSRS<br>TSESTAALGCLVKDYFPEPVTVSWNSGALTSGVHTFPAVLQSSGLYSLSSVVTVPSSNF<br>GTQTYTCNVDHKPSNTKVDKTVERKCCVECPPCPAPPVAGPSVFLFPPKPKDTLMISRT<br>PEVTCVVVDVSHEDPEVQFNWYVDGVEVHNAKTKPREEQFNSTFRVVSVLTVVHQDWLN<br>GKEYKCKVSNKGLPAPIEKTISKTKGQPREPQVYTLPPSREEMTKNQVSLTCLVKGFYP<br>SDIAVEWESNGQPENNYKTTPPMLDSDGSFFLYSKLTVDKSRWQQGNVFSCSVMHEALH<br>NHYTQKSLSLSPGK
28	anti-CD40(APX)-Flag-VH-IgG4-pCR3	MNFGFSLIFLVLVLKGVQCEVKLVPR**QL**DYKDDDDK**EF***QSLEESGGDLVKPGASLTLTC*<br>*TASGFSFSSTYVCWVRQAPGKGLEWIACIYTGDGTNYSASWAKGRFTISKPSSTTVTLQ*<br>*MTSLTPADTATYFCARPDITYGFAINFWGPGTLVTVSS***RS**SSASTKGPSVFPLAPCSRS<br>TSESTAALGCLVKDYFPEPVTVSWNSGALTSGVHTFPAVLQSSGLYSLSSVVTVPSSSL<br>GTKTYTCNVDHKPSNTKVDKRVESKYGPPCPSCPAPEFLGGPSVFLFPPKPKDTLMISR<br>TPEVTCVVVDVSQEDPEVQFNWYVDGVEVHNAKTKPREEQFNSTYRVVSVLTVLHQDWL<br>NGKEYKCKVSNKGLPSSIEKTISKAKGQPREPQVYTLPPSQEEMTKNQVSLTCLVKGFY<br>PSDIAVEWESNGQPENNYKTTPPVLDSDGSFFLYSRLTVDKSRWQEGNVFSCSVMHEAL<br>HNHYTQKSLSLSPGK
29	anti-CD40(APX)-Flag-VH-N297A-pCR3	MNFGFSLIFLVLVLKGVQCEVKLVPR**QL**DYKDDDDK**EF***QSLEESGGDLVKPGASLTLTC*<br>*TASGFSFSSTYVCWVRQAPGKGLEWIACIYTGDGTNYSASWAKGRFTISKPSSTTVTLQ*<br>*MTSLTPADTATYFCARPDITYGFAINFWGPGTLVTVSS***RS**SSASTKGPSVFPLAPSSKS<br>TSGGTAALGCLVKDYFPEPVTVSWNSGALTSGVHTFPAVLQSSGLYSLSSVVTVPSSSL<br>GTQTYICNVNHKPSNTKVDKKVEPKSCDKTHTCPPCPAPELLGGPSVFLFPPKPKDTLM<br>ISRTPEVTCVVVDVSHEDPEVKFNWYVDGVEVHNAKTKPREEQYASTYRVVSVLTVLHQ<br>DWLNGKEYKCKVSNKALPAPIEKTISKAKGQPREPQVYTLPPSRDELTKNQVSLTCLVK<br>GFYPSDIAVEWESNGQPENNYKTTPPVLDSDGSFFLYSKLTVDKSRWQQGNVFSCSVMH<br>EALHNHYTQKSLSLSPGK
30	anti-CD40(APX)-Flag-VH-N297A-scFv-anti-CD40(APX)-pCR3	MNFGFSLIFLVLVLKGVQCEVKLVPR**QL**DYKDDDDK**EF***QSLEESGGDLVKPGASLTLTC*<br>*TASGFSFSSTYVCWVRQAPGKGLEWIACIYTGDGTNYSASWAKGRFTISKPSSTTVTLQ*<br>*MTSLTPADTATYFCARPDITYGFAINFWGPGTLVTVSS***RS**SSASTKGPSVFPLAPSSKS<br>TSGGTAALGCLVKDYFPEPVTVSWNSGALTSGVHTFPAVLQSSGLYSLSSVVTVPSSSL<br>GTQTYICNVNHKPSNTKVDKKVEPKSCDKTHTCPPCPAPELLGGPSVFLFPPKPKDTLM<br>ISRTPEVTCVVVDVSHEDPEVKFNWYVDGVEVHNAKTKPREEQYASTYRVVSVLTVLHQ<br>DWLNGKEYKCKVSNKALPAPIEKTISKAKGQPREPQVYTLPPSRDELTKNQVSLTCLVK<br>GFYPSDIAVEWESNGQPENNYKTTPPVLDSDGSFFLYSKLTVDKSRWQQGNVFSCSVMH<br>EALHNHYTQKSLSLSPGK**LE***QSLEESGGDLVKPGASLTLTCTASGFSFSSTYVCWVRQA*<br>*PGKGLEWIACIYTGDGTNYSASWAKGRFTISKPSSTTVTLQMTSLTPADTATYFCARPD*<br>*ITYGFAINFWGPGTLVTVSS***RS*****STKGPKLEEGEFSEA*****QL***DIVMTQTPSSASEPVGGTVT*<br>*IKCQASQSISSRLAWYQQKPGQPPKLLIYRASTLASGVPSRFKGSGSGTEFTLTISDLE*<br>*CADAATYYCQCTGYGISWPIGGGTEVVVK*
31	anti-CD40(APX)-Flag-VH-N297A-TNC-pCR3	MNFGFSLIFLVLVLKGVQCEVKLVPR**QL**DYKDDDDK**EF***QSLEESGGDLVKPGASLTLTC*<br>*TASGFSFSSTYVCWVRQAPGKGLEWIACIYTGDGTNYSASWAKGRFTISKPSSTTVTLQ*<br>*MTSLTPADTATYFCARPDITYGFAINFWGPGTLVTVSS***RS**SSASTKGPSVFPLAPSSKS<br>TSGGTAALGCLVKDYFPEPVTVSWNSGALTSGVHTFPAVLQSSGLYSLSSVVTVPSSSL<br>GTQTYICNVNHKPSNTKVDKKVEPKSCDKTHTCPPCPAPELLGGPSVFLFPPKPKDTLM<br>ISRTPEVTCVVVDVSHEDPEVKFNWYVDGVEVHNAKTKPREEQYASTYRVVSVLTVLHQ<br>DWLNGKEYKCKVSNKALPAPIEKTISKAKGQPREPQVYTLPPSRDELTKNQVSLTCLVK<br>GFYPSDIAVEWESNGQPENNYKTTPPVLDSDGSFFLYSKLTVDKSRWQQGNVFSCSVMH<br>EALHNHYTQKSLSLSPGK**LE***DIACGCAAAPDIKDLLSRLEELEGLVSSLREQGTG*
32	anti-CD40(APX)-Flag-VL-light-pCR3	MNFGFSLIFLVLVLKGVQCEVKLVPR**QL**DYKDDDDK**EL***DIVMTQTPSSASEPVGGTVTI*<br>*KCQASQSISSRLAWYQQKPGQPPKLLIYRASTLASGVPSRFKGSGSGTEFTLTISDLEC*<br>*ADAATYYCQCTGYGISWPIGGGTEVVVK***GS**EIKRTVAAPSVFIFPPSDEQLKSGTASVV<br>CLLNNFYPREAKVQWKVDNALQSGNSQESVTEQDSKDSTYSLSSTLTLSKADYEKHKVY<br>ACEVTHQGLSSPVTKSFNRGEC
33	anti-CD40(CP-8…)-Flag-VH-IgG1(N297A+E374R/E459G/S469Y)-pCR3	MNFGFSLIFLVLVLKGVQCEVKLVPR**QL**DYKDDDDK**EF***QVQLVQSGAEVKKPGASVKVS*<br>*CKASGYTFTGYYMHWVRQAPGQGLEWMGWINPDSGGTNYAQKFQGRVTMTRDTSISTAY*<br>*MELNRLRSDDTAVYYCARDQPLGYCTNGVCSYFDYWGQGTLVTVSS***RS**SSASTKGPSVF<br>PLAPSSKSTSGGTAALGCLVKDYFPEPVTVSWNSGALTSGVHTFPAVLQSSGLYSLSSV<br>VTVPSSSLGTQTYICNVNHKPSNTKVDKKVEPKSCDKTHTCPPCPAPELLGGPSVFLFP<br>PKPKDTLMISRTPEVTCVVVDVSHEDPEVKFNWYVDGVEVHNAKTKPREEQYASTYRVV<br>SVLTVLHQDWLNGKEYKCKVSNKALPAPIEKTISKAKGQPRRPQVYTLPPSRDELTKNQ

		VSLTCLVKGFYPSDIAVEWESNGQPENNYKTTPPVLDSDGSFFLYSKLTVDKSRWQQGN VFSCSVMHGALHNHYTQKYLSLSPGK
34	anti-CD40(CP-8…)-Flag-VH-IgG1-pCR3	MNFGFSLIFLVLVLKGVQCEVKLVPR**QL**DYKDDDDK**EF***QVQLVQSGAEVKKPGASVKVS CKASGYTFTGYYMHWVRQAPGQGLEWMGWINPDSGGTNYAQKFQGRVTMTRDTSISTAY MELNRLRSDDTAVYYCARDQPLGYCTNGVCSYFDYWGQGTLVTVSS***RS**SSASTKGPSVF PLAPSSKSTSGGTAALGCLVKDYFPEPVTVSWNSGALTSGVHTFPAVLQSSGLYSLSSV VTVPSSSLGTQTYICNVNHKPSNTKVDKKVEPKSCDKTHTCPPCPAPELLGGPSVFLFP PKPKDTLMISRTPEVTCVVVDVSHEDPEVKFNWYVDGVEVHNAKTKPREEQYNSTYRVV SVLTVLHQDWLNGKEYKCKVSNKALPAPIEKTISKAKGQPREPQVYTLPPSRDELTKNQ VSLTCLVKGFYPSDIAVEWESNGQPENNYKTTPPVLDSDGSFFLYSKLTVDKSRWQQGN VFSCSVMHEALHNHYTQKSLSLSPGK
35	anti-CD40(CP-8…)-Flag-VH-IgG2-pCR3	MNFGFSLIFLVLVLKGVQCEVKLVPR**QL**DYKDDDDK**EF***QVQLVQSGAEVKKPGASVKVS CKASGYTFTGYYMHWVRQAPGQGLEWMGWINPDSGGTNYAQKFQGRVTMTRDTSISTAY MELNRLRSDDTAVYYCARDQPLGYCTNGVCSYFDYWGQGTLVTVSS***RS**SSASTKGPSVF PLAPCSRSTSESTAALGCLVKDYFPEPVTVSWNSGALTSGVHTFPAVLQSSGLYSLSSV VTVPSSNFGTQTYTCNVDHKPSNTKVDKTVERKCCVECPPCPAPPVAGPSVFLFPPKPK DTLMISRTPEVTCVVVDVSHEDPEVQFNWYVDGVEVHNAKTKPREEQFNSTFRVVSVLT VVHQDWLNGKEYKCKVSNKGLPAPIEKTISKTKGQPREPQVYTLPPSREEMTKNQVSLT CLVKGFYPSDIAVEWESNGQPENNYKTTPPMLDSDGSFFLYSKLTVDKSRWQQGNVFSC SVMHEALHNHYTQKSLSLSPGK
36	anti-CD40(CP-8…)-Flag-VH-IgG4-pCR3	MNFGFSLIFLVLVLKGVQCEVKLVPR**QL**DYKDDDDK**EF***QVQLVQSGAEVKKPGASVKVS CKASGYTFTGYYMHWVRQAPGQGLEWMGWINPDSGGTNYAQKFQGRVTMTRDTSISTAY MELNRLRSDDTAVYYCARDQPLGYCTNGVCSYFDYWGQGTLVTVSS***RS**SSASTKGPSVF PLAPCSRSTSESTAALGCLVKDYFPEPVTVSWNSGALTSGVHTFPAVLQSSGLYSLSSV VTVPSSSLGTKTYTCNVDHKPSNTKVDKRVESKYGPPCPSCPAPEFLGGPSVFLFPPKP KDTLMISRTPEVTCVVVDVSQEDPEVQFNWYVDGVEVHNAKTKPREEQFNSTYRVVSVL TVLHQDWLNGKEYKCKVSNKGLPSSIEKTISKAKGQPREPQVYTLPPSQEEMTKNQVSL TCLVKGFYPSDIAVEWESNGQPENNYKTTPPVLDSDGSFFLYSRLTVDKSRWQEGNVFS CSVMHEALHNHYTQKSLSLSPGK
37	anti-CD40(CP-8…)-Flag-VH-N297A-pCR3	MNFGFSLIFLVLVLKGVQCEVKLVPR**QL**DYKDDDDK**EF***QVQLVQSGAEVKKPGASVKVS CKASGYTFTGYYMHWVRQAPGQGLEWMGWINPDSGGTNYAQKFQGRVTMTRDTSISTAY MELNRLRSDDTAVYYCARDQPLGYCTNGVCSYFDYWGQGTLVTVSS***RS**SSASTKGPSVF PLAPSSKSTSGGTAALGCLVKDYFPEPVTVSWNSGALTSGVHTFPAVLQSSGLYSLSSV VTVPSSSLGTQTYICNVNHKPSNTKVDKKVEPKSCDKTHTCPPCPAPELLGGPSVFLFP PKPKDTLMISRTPEVTCVVVDVSHEDPEVKFNWYVDGVEVHNAKTKPREEQYASTYRVV SVLTVLHQDWLNGKEYKCKVSNKALPAPIEKTISKAKGQPREPQVYTLPPSRDELTKNQ VSLTCLVKGFYPSDIAVEWESNGQPENNYKTTPPVLDSDGSFFLYSKLTVDKSRWQQGN VFSCSVMHEALHNHYTQKSLSLSPGK
38	anti-CD40(CP-8…)-Flag-VH-N297A-TNC-pCR3	MNFGFSLIFLVLVLKGVQCEVKLVPR**QL**DYKDDDDK**EF***QVQLVQSGAEVKKPGASVKVS CKASGYTFTGYYMHWVRQAPGQGLEWMGWINPDSGGTNYAQKFQGRVTMTRDTSISTAY MELNRLRSDDTAVYYCARDQPLGYCTNGVCSYFDYWGQGTLVTVSS***RS**SSASTKGPSVF PLAPSSKSTSGGTAALGCLVKDYFPEPVTVSWNSGALTSGVHTFPAVLQSSGLYSLSSV VTVPSSSLGTQTYICNVNHKPSNTKVDKKVEPKSCDKTHTCPPCPAPELLGGPSVFLFP PKPKDTLMISRTPEVTCVVVDVSHEDPEVKFNWYVDGVEVHNAKTKPREEQYASTYRVV SVLTVLHQDWLNGKEYKCKVSNKALPAPIEKTISKAKGQPREPQVYTLPPSRDELTKNQ VSLTCLVKGFYPSDIAVEWESNGQPENNYKTTPPVLDSDGSFFLYSKLTVDKSRWQQGN VFSCSVMHEALHNHYTQKSLSLSPGK**LE***DIACGCAAAPDIKDLLSRLEELEGLVSSLRE QGTG*
39	anti-CD40(CP-8…)-Flag-VH-scFv- anti-CD40(CP-8…)-pCR3	MNFGFSLIFLVLVLKGVQCEVKLVPR**QL**DYKDDDDK**EF***QVQLVQSGAEVKKPGASVKVS CKASGYTFTGYYMHWVRQAPGQGLEWMGWINPDSGGTNYAQKFQGRVTMTRDTSISTAY MELNRLRSDDTAVYYCARDQPLGYCTNGVCSYFDYWGQGTLVTVSS***RS**SSASTKGPSVF PLAPSSKSTSGGTAALGCLVKDYFPEPVTVSWNSGALTSGVHTFPAVLQSSGLYSLSSV VTVPSSSLGTQTYICNVNHKPSNTKVDKKVEPKSCDKTHTCPPCPAPELLGGPSVFLFP PKPKDTLMISRTPEVTCVVVDVSHEDPEVKFNWYVDGVEVHNAKTKPREEQYASTYRVV SVLTVLHQDWLNGKEYKCKVSNKALPAPIEKTISKAKGQPREPQVYTLPPSRDELTKNQ VSLTCLVKGFYPSDIAVEWESNGQPENNYKTTPPVLDSDGSFFLYSKLTVDKSRWQQGN VFSCSVMHEALHNHYTQKSLSLSPGK**LE***QVQLVQSGAEVKKPGASVKVSCKASGYTFTG YYMHWVRQAPGQGLEWMGWINPDSGGTNYAQKFQGRVTMTRDTSISTAYMELNRLRSDD TAVYYCARDQPLGYCTNGVCSYFDYWGQGTLVTVSS***RS*****STKGPKLEEGEFSEAQL***DIQM TQSPSSVSASVGDRVTITCRASQGIYSWLAWYQQKPGKAPNLLIYTASTLQSGVPSRFS GSGSGTDFTLTISSLQPEDFATYYCQQANIFPLTFGGGTKVEIK*
40	anti-CD40(CP-8…)-Flag-VL-light-pCR3	MNFGFSLIFLVLVLKGVQCEVKLVPR**QL**DYKDDDDK**EL***DIQMTQSPSSVSASVGDRVTI TCRASQGIYSWLAWYQQKPGKAPNLLIYTASTLQSGVPSRFSGSGSGTDFTLTISSLQP EDFATYYCQQANIFPLTFGGGTKVEIK***GS**EIKRTVAAPSVFIFPPSDEQLKSGTASVVC LLNNFYPREAKVQWKVDNALQSGNSQESVTEQDSKDSTYSLSSTLTLSKADYEKHKVYA CEVTHQGLSSPVTKSFNRGEC
41	anti-CD40(V12t)-Fc(DANA)-Flag-pCR3	MNFGFSLIFLVLVLKGVQCEVKLVPR**QL***QVQLQESGGGLVQAGGSLRLSCAASGLVFKR YSMNWYRQPPGQQRGLVASISDSGVSTNYADSVKGRFTISRDNAKNIGYLQMNSLKPED TAVYYCNMHTFWGQGTQVTVSS***GS**KTHTCPPCPAPELLGGPSVFLFPPKPKDTLMISRT PEVTCVVVAVSHEDPEVKFNWYVDGVEVHNAKTKPREEQYASTYRVVSVLTVLHQDWLN GKEYKCKVSNKALPAPIEKTISKAKGQPREPQVYTLPPSRDELTKNQVSLTCLVKGFYP SDIAVEWESNGQPENNYKTTPPVLDSDGSFFLYSKLTVDKSRWQQGNVFSCSVMHEALH NHYTQKSLSLSPGK**EF**DYKDDDDK**LE**
42	anti-CD40(V12t)-VHH-Flag-TNC-pCR3	MNFGFSLIFLVLVLKGVQCEVKLVPR**QL**QVQLQESGGGLVQAGGSLRLSCAASGLVFKR YSMNWYRQPPGQQRGLVASISDSGVSTNYADSVKGRFTISRDNAKNIGYLQMNSLKPED TAVYYCNMHTFWGQGTQVTVSS**GS**DYKDDDDK*DIACGCAAAPDIKDLLSRLEELEGLVS SFREQGTG*

43	anti-CD40(V12t)-VHH-Flag-N297A-pCR3	MNFGFSLIFLVLVLKGVQCEVKLVPR**QL**DYKDDDDK**EL**QVQLQESGGGLVQAGGSLRLSCAASGLVFKRYSMNWYRQPPGQQRGLVASISDSGVSTNYADSVKGRFTISRDNAKNIGYLQMNSLKPEDTAVYYCNMHTFWGQGTQVTVSS**GS**SSASTKGPSVFPLAPSSKSTSGGTAALGCLVKDYFPEPVTVSWNSGALTSGVHTFPAVLQSSGLYSLSSVVTVPSSSLGTQTYICNVNHKPSNTKVDKKVEPKSCDKTHTCPPCPAPELLGGPSVFLFPPKPKDTLMISRTPEVTCVVVDVSHEDPEVKFNWYVDGVEVHNAKTKPREEQYASTYRVVSVLTVLHQDWLNGKEYKCKVSNKALPAPIEKTISKAKGQPREPQVYTLPPSRDELTKNQVSLTCLVKGFYPSDIAVEWESNGQPENNYKTTPPVLDSDGSFFLYSKLTVDKSRWQQGNVFSCSVMHEALHNHYTQKSLSLSPGK
44	anti-CD40(V12t)-VHH-Flag-light-pCR3	MNFGFSLIFLVLVLKGVQCEVKLVPR**QL**DYKDDDDK**EL**QVQLQESGGGLVQAGGSLRLSCAASGLVFKRYSMNWYRQPPGQQRGLVASISDSGVSTNYADSVKGRFTISRDNAKNIGYLQMNSLKPEDTAVYYCNMHTFWGQGTQVTVSS**GS**EIKRTVAAPSVFIFPPSDEQLKSGTASVVCLLNNFYPREAKVQWKVDNALQSGNSQESVTEQDSKDSTYSLSSTLTLSKADYEKHKVYACEVTHQGLSSPVTKSFNRGEC
45	anti-CD40(V12t)-VHH(3x)-Fc(DANA)-Flag-pCR3	MNFGFSLIFLVLVLKGVQCEVKLVPR**GT**QVQLQESGGGLVQAGGSLRLSCAASGLVFKRYSMNWYRQPPGQQRGLVASISDSGVSTNYADSVKGRFTISRDNAKNIGYLQMNSLKPEDTAVYYCNMHTFWGQGTQVTVSS**RSGGGGSGGGGSGGGGSGGGGSGGGGS**QVQLQESGGGLVQAGGSLRLSCAASGLVFKRYSMNWYRQPPGQQRGLVASISDSGVSTNYADSVKGRFTISRDNAKNIGYLQMNSLKPEDTAVYYCNMHTFWGQGTQVTVSS**GGGGSGGGGSGGGGSGGGGSGGGGSQL**QVQLQESGGGLVQAGGSLRLSCAASGLVFKRYSMNWYRQPPGQQRGLVASISDSGVSTNYADSVKGRFTISRDNAKNIGYLQMNSLKPEDTAVYYCNMHTFWGQGTQVTVSS**GS**KTHTCPPCPAPELLGGPSVFLFPPKPKDTLMISRTPEVTCVVVAVSHEDPEVKFNWYVDGVEVHNAKTKPREEQYASTYRVVSVLTVLHQDWLNGKEYKCKVSNKALPAPIEKTISKAKGQPREPQVYTLPPSRDELTKNQVSLTCLVKGFYPSDIAVEWESNGQPENNYKTTPPVLDSDGSFFLYSKLTVDKSRWQQGNVFSCSVMHEALHNHYTQKSLSLSPGK**EF**DYKDDDDK**LE**
46	anti-CD40(V12t)(3x)-Flag-TNC-pCR3	MNFGFSLIFLVLVLKGVQCEVKLVPR**GT**QVQLQESGGGLVQAGGSLRLSCAASGLVFKRYSMNWYRQPPGQQRGLVASISDSGVSTNYADSVKGRFTISRDNAKNIGYLQMNSLKPEDTAVYYCNMHTFWGQGTQVTVSS**RSGGGGSGGGGSGGGGSGGGGSGGGGS**QVQLQESGGGLVQAGGSLRLSCAASGLVFKRYSMNWYRQPPGQQRGLVASISDSGVSTNYADSVKGRFTISRDNAKNIGYLQMNSLKPEDTAVYYCNMHTFWGQGTQVTVSS**GGGGSGGGGSGGGGSGGGGSGGGGSQL**QVQLQESGGGLVQAGGSLRLSCAASGLVFKRYSMNWYRQPPGQQRGLVASISDSGVSTNYADSVKGRFTISRDNAKNIGYLQMNSLKPEDTAVYYCNMHTFWGQGTQVTVSS**GS**DYKDDDDKDIACGCAAAPDIKDLLSRLEELEGLVSSLREQGTG
47	CD40(ed)-2xFlag-Gaussia(w/o)-pCR3	MVRLPLQCVLWGCLLTAVHPEPPTACREKQYLINSQCCSLCQPGQKLVSDCTEFTETECLPCGESEFLDTWNRETHCHQHKYCDPNLGLRVQQKGTSETDTICTCEEGWHCTSEACESCVLHRSCSPGFGVKQIATGVSDTICEPCPVGFFSNVSSAFEKCHPWTSCETKDLVVQQAGTNKTDVVCGPQDRL**GS**DYKDDDDK**EF**DYKDDDDK**LE**KPTENNEDFNIVAVASNFATTDLDADRGKLPGKKLPLEVLKEMEANARKAGCTRGCLICLSHIKCTPKMKKFIPGRCHTYEGDKESAQGGIGEAIVDIPEIPGFKDLEPMEQFIAQVDLCVDCTTGCLKGLANVQCSDLLKKWLPQRCATFASKIQGQVDKIKGAGGD
48	CD40-CRD1,2,3,4-2xFlag-Gaussia(w/o)-pCR3	MVRLPLQCVLWGCLLTAVHPEPPTACREKQYLINSQCCSLCQPGQKLVSDCTEFTETECLPCGESEFLDTWNRETHCHQHKYCDPNLGLRVQQKGTSETDTICTCEEGWHCTSEACESCVLHRSCSPGFGVKQIATGVSDTICEPCPVGFFSNVSSAFEKCHPWTSCETKDLVVQQAGTNKTDVVCG**GS**DYKDDDDK**EF**DYKDDDDK**LE**KPTENNEDFNIVAVASNFATTDLDADRGKLPGKKLPLEVLKEMEANARKAGCTRGCLICLSHIKCTPKMKKFIPGRCHTYEGDKESAQGGIGEAIVDIPEIPGFKDLEPMEQFIAQVDLCVDCTTGCLKGLANVQCSDLLKKWLPQRCATFASKIQGQVDKIKGAGGD
49	CD40-CRD1,2,3-2xFlag-Gaussia(w/o)-pCR3	MVRLPLQCVLWGCLLTAVHPEPPTACREKQYLINSQCCSLCQPGQKLVSDCTEFTETECLPCGESEFLDTWNRETHCHQHKYCDPNLGLRVQQKGTSETDTICTCEEGWHCTSEACESCVLHRSCSPGFGVKQIATGVSDTICE**GS**DYKDDDDK**EF**DYKDDDDK**LE**KPTENNEDFNIVAVASNFATTDLDADRGKLPGKKLPLEVLKEMEANARKAGCTRGCLICLSHIKCTPKMKKFIPGRCHTYEGDKESAQGGIGEAIVDIPEIPGFKDLEPMEQFIAQVDLCVDCTTGCLKGLANVQCSDLLKKWLPQRCATFASKIQGQVDKIKGAGGD
50	CD40-CRD1-2-2xFlag-Gaussia(w/o)-pCR3	MVRLPLQCVLWGCLLTAVHPEPPTACREKQYLINSQCCSLCQPGQKLVSDCTEFTETECLPCGESEFLDTWNRETHCHQHKYCDPNLGLRVQQKGTSETDTIC**GS**DYKDDDDK**EF**DYKDDDDK**LE**KPTENNEDFNIVAVASNFATTDLDADRGKLPGKKLPLEVLKEMEANARKAGCTRGCLICLSHIKCTPKMKKFIPGRCHTYEGDKESAQGGIGEAIVDIPEIPGFKDLEPMEQFIAQVDLCVDCTTGCLKGLANVQCSDLLKKWLPQRCATFASKIQGQVDKIKGAGGD
51	CD40-CRD1-2xFlag-Gaussia(w/o)-pCR3	MVRLPLQCVLWGCLLTAVHPEPPTACREKQYLINSQCCSLCQPGQKLVSDCTEFTETECL**GS**DYKDDDDK**EF**DYKDDDDK**LE**KPTENNEDFNIVAVASNFATTDLDADRGKLPGKKLPLEVLKEMEANARKAGCTRGCLICLSHIKCTPKMKKFIPGRCHTYEGDKESAQGGIGEAIVDIPEIPGFKDLEPMEQFIAQVDLCVDCTTGCLKGLANVQCSDLLKKWLPQRCATFASKIQGQVDKIKGAGGD

Supplemental Table S2. Plasmids used (see supplemental table S1) for production of antibody variants.

Proteine name	Plasmids(s)
αCD40(G28.5)-IgG1	2 + 8
αCD40(ADC)-IgG1	19 + 24
αCD40(APX)-IgG1	26 + 32
αCD40(ChiLob)-IgG1	13 + 16

αCD40(CP8-…)-IgG1	34 + 40
αCD40(G28.5)-IgG2	3 + 8
αCD40(ADC)-IgG2	20 + 24
αCD40(APX)-IgG2	27 + 32
αCD40(ChiLob)-IgG2	14 + 16
αCD40(CP8-…)-IgG2	35 + 40
αCD40(G28.5)-IgG4	4 + 8
αCD40(ADC)-IgG4	21 + 24
αCD40(APX)-IgG4	28 + 32
αCD40(ChiLob)-IgG4	15 + 16
αCD40(CP8-…)-IgG4	36 + 40
αCD40(G28.5)-IgG1(N297A)	1 + 8
αCD40(ADC)-IgG1(N297A)	17 + 24
αCD40(APX)-IgG1(N297A)	29 + 32
αCD40(ChiLob)-IgG1(N297A)	10 + 16
αCD40(CP8-…)-IgG1(N297A)	37 + 40
αCD40(G28.5)-IgG1(N297A)-HC:scFv(G28.5)	7 + 8
αCD40(ADC)-IgG1(N297A)-HC:scFv(ADC)	22 + 24
αCD40(APX)-IgG1(N297A)-HC:scFv(APX)	30 + 32
αCD40(ChiLob)-IgG1(N297A)-HC:scFv(ChiLob)	11 + 16
αCD40(CP8-…)-IgG1(N297A)-HC:scFv(CP8-…)	39 + 40
αCD40(G28.5)-IgG1(N297A)-HC:TNC	5 + 8
αCD40(ADC)-IgG1(N297A)-HC:TNC	23 + 24
αCD40(APX)-IgG1(N297A)-HC:TNC	31 + 32
αCD40(ChiLob)-IgG1(N297A)-HC:TNC	12 + 16
αCD40(CP8-…)-IgG1(N297A)-HC:TNC	38 + 40
αCD40(G28.5)-IgG1(N297A)-RGY	6 + 8
αCD40(ADC)-IgG1(N297A)-RGY	18 + 24
αCD40(APX)-IgG1(N297A)-RGY	25 + 32
αCD40(ChiLob)-IgG1(N297A)-RGY	9 + 16
αCD40(CP8-…)-IgG1(N297A)-RGY	33 + 40
V12t-CH/V12t-CL	43 + 44

Supplemental Table S3. Sources of amino acid sequences.

	**Amino acids**	**GenBank (accesion)**
Signalpeptide	MNFGFSLIFLVLVLKGVQCEVKLVPR	
Flag tag	DYKDDDDK	
Tenascin-C	110-139	AAA49086.1
Restriction sites	QL encoded by Mfe1 (CAATTG)<br>EL encoded by EcoR1/Mfe1 (GAATTG)<br>GS encoded by BamH1 (GGATCC)<br>EF encoded by EcoR1 (GAATTC)<br>LE encoded by Xho1 (CTCGAG)	
Gaussia princeps luciferase (GpL)	18-185	AAG54095
Constant heavy chain of human IgG1	145-476	AAA02914.1

Constant heavy chain of human IgG1(A-RGY), with Fc mutation N287A/E374R/E459G/S469Y[1]	145-476 with Fc mutation N287A/E374R/E459G/S469Y[1]	AAA02914.1
Constant heavy chain of human IgG1(N297A), with Fc mutation N287A[1]	145-476 with Fc mutation N287A[1]	AAA02914.1
Constant heavy chain of human IgG2	1-325	AAB59393.1
Constant heavy chain of human IgG4	1-326	AAB59394.1
Constant light chain	105-214	BAA97671.1
Linker	GGGGSGGGGSGGGGSGGGGSGGGGS	
CD40(ed)	1-192	AAO43990.1
CD40(ed)-CRD1-4	1-187	AAO43990.1
CD40(ed)-CRD1-3	1-144	AAO43990.1
CD40(ed)-CRD1-2	1-103	AAO43990.1
CD40(ed)-CDR1	1-60	AAO43990.1
VH and VL of G28.5 domain	VL: 148-259 VH: 21-130 (from scFv sequence)	AJ853736
Selicrelumab	VL: 1-117, VH: 1-126	KEGG drug database entry D11491
APX005M	As indicated VL and VH in aa sequence for light and heavy chain R-8	WO 2014/070934 Al
ChiLob7.4	VL: 1-117, VH: 1-122	PDB entries 6FAX_L (V L) and 6FAX_H (VH)
Mitazalimab	VL: 1-110, VH: 1-119	Thera-SAbDab[2]
V12t	1-112	De Weerdt et al., 2021[3]

[1] The numbering of N297 as well as of E374, E459 and S469 in the N297A and E374R/E459G/S469Y mutations refers to that given in the original publications (ref. 36 and 39) and are not identical with the corresponding numbering in the Genbank sequence.

[2] Therapeutic Structural Antibody Database

[3] de Weerdt I, Lameris R, Scheffer GL, Vree J, de Boer R, Stam AG, et al. A Bispecific Antibody Antagonizes Prosurvival CD40 Signaling and Promotes Vγ9Vδ2 T cell-Mediated Antitumor Responses in Human B-cell Malignancies. Cancer Immunol Res. 2021; 9: 50-61.
